# RCN1 induces sorafenib resistance and malignancy in hepatocellular carcinoma by activating c-MYC signaling via the IRE1α–XBP1s pathway

**DOI:** 10.1038/s41420-021-00696-6

**Published:** 2021-10-18

**Authors:** Jia-Wei Wang, Li Ma, Yuan Liang, Xiao-Jun Yang, Song Wei, Hao Peng, Shi-Pei Qiu, Xu Lu, Ya-Qing Zhu, Bao-Lin Wang

**Affiliations:** 1 Department of General Surgery, The Second Affiliated Hospital of Nanjing Medical University, 121 Jiangjiayuan Road, 210011 Nanjing, Jiangsu China; 2 Cancer Medical Center, The Second Affiliated Hospital of Nanjing Medical University, 121 Jiangjiayuan Road, 210011 Nanjing, Jiangsu China; 3 School of Biomedical Engineering and Informatics, Nanjing Medical University, Nanjing, China; 4 School of Medicine, Southeast University, Nanjing, China; 5 Department of Hepatobiliary Surgery, the First Affiliated Hospital of Guangzhou University of Traditional Chinese Medicine, 16 Jichang Road, Baiyun District, 510405 Guangzhou, China

**Keywords:** Apoptosis, Stress signalling, Oncogenes

## Abstract

The increasing incidence of hepatocellular carcinoma (HCC) is of great concern globally, but the molecular pathogenesis of these tumors remains unclear. Sorafenib is a first-line drug for the treatment of advanced HCC. However, the efficacy of sorafenib in improving patient survival is limited, and most patients inevitably develop resistance to this drug. Recent studies have demonstrated that the activation of the IRE1α–XBP1s pathway might play a protective role in the response to sorafenib and contribute to malignancy in HCC. Here, we found that RCN1, an endoplasmic reticulum resident protein, is significantly upregulated in sorafenib-resistant HCC cells and promotes tumor progression. Our analysis showed that RCN1 may be an independent predictor of tumor recurrence and overall survival. Mechanistically, RCN1 promotes the dissociation of GRP78 from IRE1α in sorafenib-resistant cells by interacting with GRP78 through its EFh1/2 domain. Subsequently, the IRE1α–XBP1s pathway, a branch of the unfolded protein response, is sustainably activated. Interestingly, IRE1α–XBP1s pathway activity is required for c-MYC signaling, one of the most highly activated oncogenic pathways in HCC. These results suggest that RCN1-targeted therapy might be a feasible strategy for the treatment of HCC.

## Introduction

Liver cancer is the sixth most commonly diagnosed cancer worldwide (4.7% of all cases), and its fatality rate (8.3%) is the third highest among malignant tumors in men and women combined. Hepatocellular carcinoma (HCC) is the most common type of primary liver cancer, comprising 75–85% of all liver cancer cases [1]. Therefore, there is a great need for a better understanding of the mechanisms underlying HCC pathogenesis.

Sorafenib, an oral multi-kinase inhibitor, exerts anti-angiogenic and anti-proliferative effects by repressing serine/threonine kinases and receptor tyrosine kinases [2]. Currently, sorafenib is the first-line standard treatment for advanced HCC, and it demonstrates an obvious curative effect. Sorafenib can extend the median overall survival of patients with advanced-stage HCC from 8 to 11 months [3]. However, the benefits of sorafenib are limited. Only ~30% of patients benefit from the drug, but they too acquire resistance within 6 months, suggesting the existence of primary and acquired sorafenib resistance in HCC [4]. Therefore, research on molecular regulation in sorafenib-resistant HCC cells is urgently warranted.

Sorafenib can induce endoplasmic reticulum (ER) stress-related apoptosis in HCC. Subsequently, the unfolded protein response (UPR) — a well-defined process that plays a vital role in restoring homeostasis after the accumulation of potentially toxic misfolded proteins — is required to restore ER homeostasis [5, 6]. In response to chronic stress, some cancer cells constitutively activate the UPR pathway and become resistant to cell death [7]. IRE1α, a UPR sensor, can protect cells against ER stress [5]. Studies have demonstrated that the IRE1α–XBP1s pathway is important for tumor survival under sorafenib-induced ER stress [8]. Sustained activation of IRE1α–XBP1s signaling not only confers sorafenib resistance to HCC cells but also contributes to tumorigenesis and the epithelial–mesenchymal transition (EMT) [9, 10]. Hence, it is necessary to investigate the role of the UPR, and especially that of the IRE1α–XBP1s pathway, in sorafenib resistance in HCC. A previous study found that the IRE1α–XBP1s pathway can directly activate c-MYC signaling [11]. Moreover, MYC dysregulation is commonly observed in multiple human cancers, including HCC [12]. Further, c-MYC signaling plays an important role in drug resistance [13]. Unfortunately, owing to its “undruggable” protein structure, there is no specific therapy that directly destroys MYC function. Therefore, identifying the key genes upstream of MYC can help in developing alternative strategies for the treatment of HCC, and especially sorafenib-resistant HCC.

Reticulocalbin 1 (RCN1) is a member of the CREC family. This protein, which is located in the ER, consists of an ER-retention motif, HDEL, and six EF-hand motifs in its carboxyl-terminal sequence. This Ca2^+^ binding protein is involved in the regulation of Ca2^+^-dependent activity in the ER lumen and is present throughout the secretory pathway in mammalian cells [14]. RCN1 overexpression has been identified in various tumors, including liver, lung, breast, colorectal, prostate, and nasopharyngeal cancers [14–20]. In particular, RCN1 was implicated in the regulation of drug resistance, and RCN1 knockdown was found to reduce the resistance of nasopharyngeal carcinoma (NPC) cells/tissues to doxorubicin, promoting NPC cell death [21]. RCN1 was also found to be upregulated in doxorubicin-resistant uterine cancer [22]. However, the exact mechanisms of RCN1-mediated sorafenib resistance and tumorigenesis in HCC are unclear.

Therefore, in the present study, we aimed to examine whether RCN1 participates in sorafenib resistance and hepatocarcinogenesis in HCC and to clarify the molecular mechanism of its role in this type of cancer.

## Results

### RCN1 is upregulated in sorafenib-resistant HCC cells and may predict poor patient prognosis

**Fig. 1:**
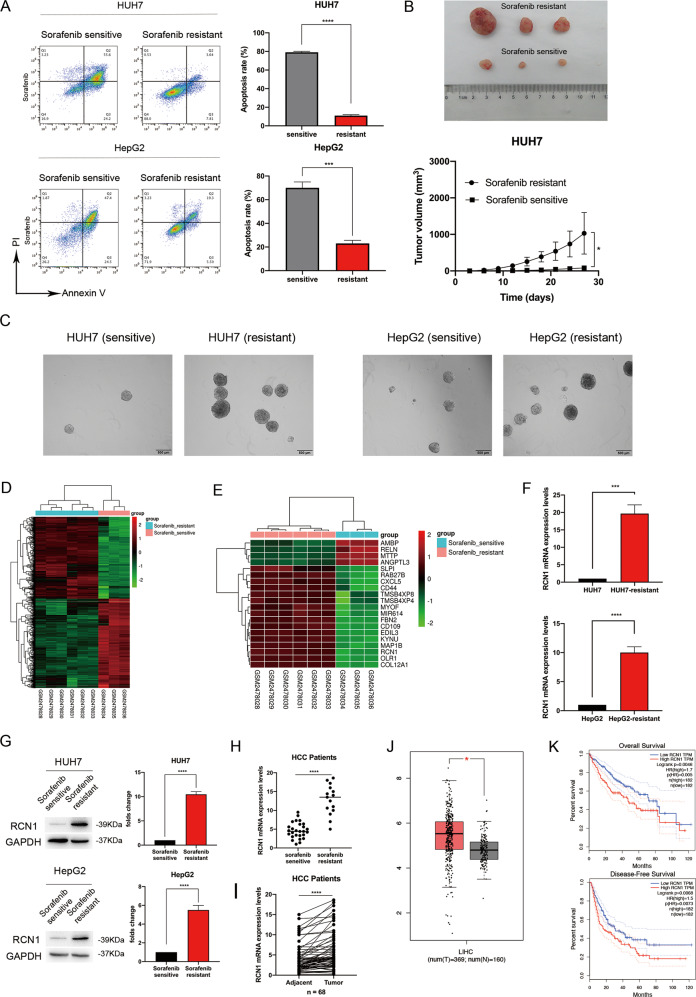
RCN1 is upregulated in sorafenib-resistant HCC cells and may predict a poor prognosis. **A** Representative flow cytometry analysis of Annexin V-PI staining in Huh7 and HepG2 sorafenib-sensitive and -resistant cells treated with 5 μM sorafenib. **B** Subcutaneous injection of sorafenib-sensitive and -resistant Huh7 cells into nude mice, and ex vivo images of resected xenografts. **C** Self-renewal ability of sorafenib-resistant HCC cells examined using the sphere formation assay. **D** Heatmap of 1145 genes differentially expressed between sorafenib-sensitive cells and cells with acquired sorafenib resistance from the GSE94550 dataset. **E** Heatmap of the 20 most variable genes (|logFC|>3). The abscissa in panels **A** and **B** represents the sample number, the ordinate represents the differentially expressed genes, the histogram at the upper right represents the color level, and each cell in the matrix represents a specific expression level. **F** Genomic RCN1 expression in Huh7 and HepG2 sorafenib-sensitive and -resistant cells. **G** Proteomic RCN1 expression in Huh7 and HepG2 sorafenib-sensitive and -resistant cells. **H** RCN1 expression was upregulated in sorafenib-resistant HCC when compared with sorafenib-sensitive HCC. **I** *RCN1* mRNA expression was upregulated in HCC tumor tissues (*n* = 68) compared with the corresponding adjacent tissues (*n* = 68). *GAPDH* served as an internal reference. **J** RCN1 expression was significantly higher in tumor tissues than in the adjacent tissues, according to the GEPIA website. **K** Association of RCN1 expression with overall survival (top) and disease-free survival (bottom). Data are presented as the means ± SEM of three independent experiments; data for western blot have undergone quantitative analysis. ns not significantly different. *P < 0.05; **P < 0.01; ***P < 0.001; ****P < 0.0001, *t*-test.

We calculated the half maximal inhibitory concentration (IC50) of sorafenib in the resistant cell lines using the MTT test (Fig. S1A). Using the annexin V-propidium iodide (PI) assay, we found that sorafenib-resistant cells had a greater ability to resist sorafenib-induced apoptosis (Fig. 1A). Moreover, sorafenib-resistant cells had a greater capability of initiating tumor formation in vivo (Fig. 1B). More importantly, the diameter and numbers of tumors were larger in the sorafenib resistance group (Fig. 1C). These data demonstrated the successful establishment of sorafenib-resistant cell lines.

The GSE94550 dataset, which contains information on sorafenib resistance in HCC, was analyzed using the R language. We found 1145 genes differentially expressed between cells that were sensitive to sorafenib and those that had acquired resistance to the drug (Fig. 1D). Gene ontology (GO) enrichment, Kyoto Encyclopedia of Genes and Genomes (KEGG) pathway analysis, and gene set enrichment analysis (GSEA) were performed to further examine the functional alterations associated with sorafenib resistance (Fig. S1B–D). Subsequently, we created a heatmap of the 20 genes showing the highest fold change (|logFC|> 3), as illustrated in Fig. 1E. Using qRT-PCR, we confirmed that *RCN1*, one of the genes with the highest fold change, was significantly upregulated in sorafenib-resistant Huh7 and HepG2 cells (Fig. 1F). We also verified that RCN1 protein levels were indeed elevated in sorafenib-resistant cells (Fig. 1G). Then, we evaluated RCN1 expression in sorafenib-resistant (*n* = 15) and sorafenib-sensitive (*n* = 27) HCC samples from patients (Fig. 1H). The clinical characteristics of sorafenib-resistant patients are summarized in Table 1. To further elucidate the role of RCN1 in HCC, we examined RCN1 expression in HCC tumor tissues and matched tumor-adjacent tissues (*n* = 68) (Fig. 1I). We observed that high levels of RCN1 were significantly associated with a larger tumor size, microvascular invasion and higher TNM stage (Table 2). Using the GEPIA website, we found that RCN1 expression was significantly higher in tumor tissues than in the adjacent tissues (Fig. 1J). We analyzed the effect of RCN1 expression on the prognosis of HCC patients and found that patients with a high expression of RCN1 had a worse prognosis (Fig. 1K). Taken together, these data suggested that RCN1 could be a potential prognostic marker in HCC patients, and further basic research on this topic was warranted.

**Table 1:** Relationship between sorafenib resistance and intratumoral RCN1 expression, clinicopathologic features (*n* = 42).

No. (%) of patients					
Characteristics	Total	Sorafenib-sensitive	Sorafenib-resistant	*χ*2	*p*-value
RCN1 expression				8.849	0.003^a^
Low	24	20 (74.1)	4 (26.8)	–	–
High	18	7 (25.9)	11 (73.2)	–	–
Age (y)				0.616	0.433
<50	11	6 (22.2)	5 (33.3)	–	–
≥50	31	21 (77.8)	10 (66.7)	–	–
Gender				2.696	0.101
Male	29	21 (77.8)	8 (53.3)	–	–
Female	13	6 (22.2)	7 (46.7)	–	–
Cirrhosis				0.305	0.580
Present	22	15 (55.6)	7 (46.6)	–	–
Absent	20	12 (44.4)	8 (53.4)	–	–
HBV infection				0.017	0.895
Positive	36	23 (85.2)	13 (86.7)	–	–
Negative	6	4 (14.8)	2 (13.3)	–	–
Tumor size (cm)				10.996	0.001^a^
<5	20	18 (66.7)	2 (13.3)	–	–
≥5	22	9 (33.3)	13 (86.7)	–	–
Vascular invasion				7.135	0.008^a^
Presence	25	12 (44.4)	13 (86.7)	–	–
Absence	17	15 (55.6)	2 (13.3)	–	–
AFP (ng/ml)				0.494	0.482
≤20	23	15 (55.6)	8 (53.4)	–	–
>20	19	12 (44.4)	7 (46.6)	–	–
TNM stage				6.067	0.014^a^
I	16	14 (51.9)	2 (13.3)	–	–
II + III	26	13 (48.1)	13 (86.7)	–	–

^a^*p*-value < 0.05.

**Table 2:** Association between RCN1 expression and clinicopathological features in patients with HCC (*n* = 68).

No. (%) of patients					
Characteristics	Total	Low-RCN1	High-RCN1	*χ*^2^	*p*-value
Age (y)				2.138	0.144
<50	15	5 (14.7)	10 (29.4)	–	–
≥50	53	29 (85.3)	24 (70.6)	–	–
Gender				1.722	0.189
Male	47	26 (76.5)	21 (61.8)	–	–
Female	21	8 (23.5)	13 (38.2)	–	–
Cirrhosis				0.236	0.627
Present	32	15 (44.1)	17 (50.0)	–	–
Absent	36	19 (55.9)	17 (50.0)	–	–
HBV infection				0.108	0.742
Positive	57	29 (85.3)	28 (82.4)	–	–
Negative	11	5 (14.7)	6 (17.6)	–	–
Tumor size (cm)				11.691	0.001^a^
<5	38	26 (76.5)	12 (35.3)		
≥5	30	8 (23.5)	22 (64.7)		
Vascular invasion				8.500	0.004^a^
Presence	35	11 (32.4)	24 (70.6)	–	–
Absence	33	23 (67.6)	10 (29.4)	–	–
AFP (ng/ml)				0.541	0.462
≤20	29	13 (38.2)	16 (47.1)	–	–
>20	39	21 (61.8)	18 (52.9)	–	–
TNM stage				13.339	0.000^a^
I	31	23 (67.6)	8 (23.5)	–	–
II + III	37	11 (32.4)	26 (76.5)	–	–

^a^*p*-value < 0.05.

### RCN1 promotes sorafenib resistance and HCC malignancy

**Fig. 2:**
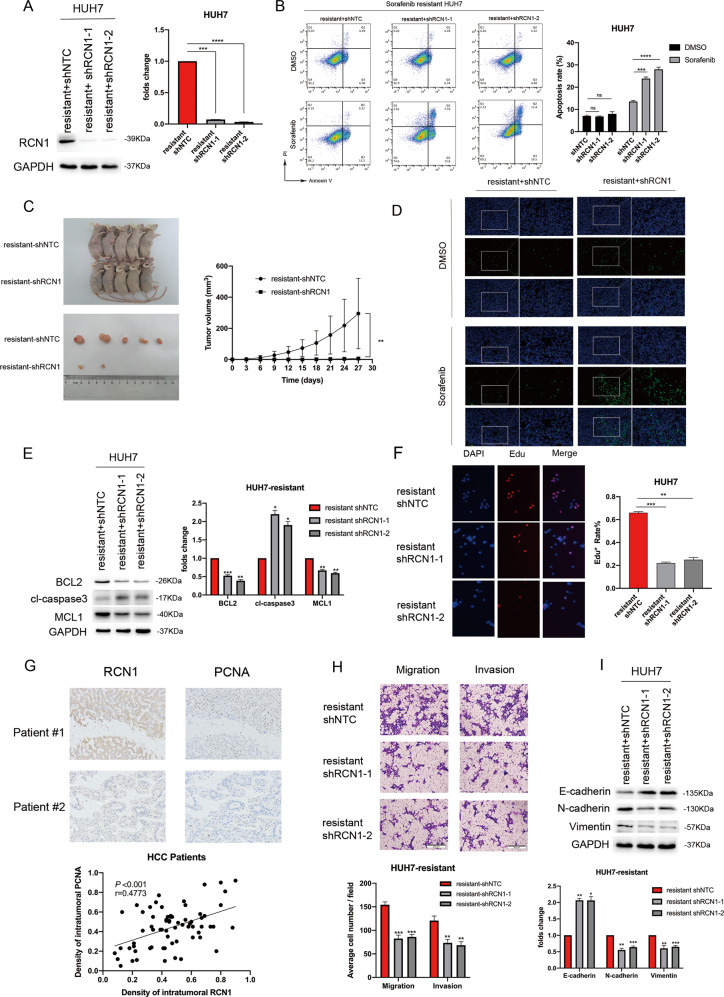
RCN1 contributes to sorafenib resistance and HCC malignancy. **A** RCN1 protein expression in Huh7 sorafenib-resistant cells after RCN1 knockdown. **B** Representative flow cytometry analysis of Annexin V-PI staining in Huh7 sorafenib-resistant cells with or without RCN1 silencing, in the presence of 5 μM sorafenib. **C** Sorafenib-resistant Huh7 cells with and without RCN1 silencing were subcutaneously injected into the flanks of NOD/SCID mice. The images of harvested tumors (*n* = 5/group) and tumor growth curves are presented. **D** TUNEL staining for apoptotic cells in tumor xenografts. **E** Expression of apoptosis-related proteins in sorafenib-resistant Huh7 cells with or without stably downregulated RCN1. **F** Cell proliferation in sorafenib-resistant Huh7 cells with RCN1 knockdown assessed using an EdU assay. **G** RCN1 expression was positively correlated with that of PCNA in clinical tumor tissues (*n* = 68). **H** Transwell assays of cell migration and invasion in sorafenib-resistant Huh7 cells after RCN1 knockdown. **I** Western blot analysis of RCN1, E-cadherin, N-cadherin, and Vimentin expression in sorafenib-resistant Huh7 cells with or without stably downregulated RCN1. Data are presented as the means ± SEM of three independent experiments; data for western blot have undergone quantitative analysis. *P < 0.05; **P < 0.01; ***P < 0.001; ****P < 0.0001, *t*-test.

Among the five sorafenib-sensitive HCC cell lines — i.e., Huh7, HepG2, MHCC-LM3, MHCC-97H, and Hep3B — the expression of RCN1 was the lowest in Huh7 cells and the highest in Hep3B cells (Fig. S2A). Further, RCN1 overexpression in Huh7 cells resulted in an enhanced ability to resist sorafenib-induced apoptosis, whereas the knockdown of RCN1 in Hep3B cells led to a decline in resistance (Fig. S2B, C). These findings confirmed the link between RCN1 expression and sorafenib resistance. In order to explore the role of RCN1 in sorafenib resistance, we knocked down RCN1 in sorafenib-resistant Huh7 cells (Fig. 2A). RCN1 silencing reduced the ability of sorafenib-resistant cells to resist sorafenib-induced apoptosis (Fig. 2B). Moreover, when we subcutaneously injected sorafenib-resistant Huh7 cells into sorafenib-treated nude mice, RCN1 knockdown significantly enhanced the effectiveness of sorafenib against HCC (Fig. 2C). Subsequently, when a terminal deoxynucleotidyl transferase dUTP nick end labeling (TUNEL) assay was performed, a higher percentage of apoptotic cells was observed in resected RCN1-knockdown xenografts (Fig. 2D). In addition, the expression of apoptosis-related proteins also significantly changed after RCN1 knockdown, further demonstrating that RCN1 promoted apoptosis resistance (Fig. 2E).

Data from the TIMER2.0 website showed a positive correlation between the expressions of RCN1 and proliferating cell nuclear antigen (PCNA) (Fig. S2D). The CCK-8 assay further revealed that RCN1 knockdown reduced proliferation in sorafenib-resistant cells (Fig. S2E). Consistent with this, EdU incorporation assays also showed that RCN1 knockdown resulted in a significant inhibition of the proliferation of sorafenib-resistant cells (Fig. 2F). Examination of clinical tumor tissues using IHC also proved that the expression of RCN1 was positively correlated with that of PCNA (*n* = 68) (Fig. 2G).

Further, transwell assays showed that RCN1 knockdown reduced the migratory and invasive capacities of sorafenib-resistant cells (Fig. 2H). We hypothesized that RCN1 played a role in EMT, and we further investigated the correlation between the expression of EMT markers and that of RCN1 using the TIMER2.0 website (Fig. S2F). We found that high RCN1 expression inhibited E-cadherin levels and augmented vimentin and N-cadherin levels in sorafenib-resistant cells (Fig. 2I).

### RCN1 contributes to selective activation of the unfolded protein response

We speculated that the role of RCN1 in sorafenib-resistant cells may be related to ER stress. Transmission electron microscopy revealed that sorafenib-resistant cells had an irregular ER structure, with substantially expanded membranes and distended lumens when compared with sorafenib-resistant shRCN1 cells (Fig. S3A). Previous studies have shown that RCN1 can resist tunicamycin (TM)-induced apoptosis in HepG2 cells by inhibiting the PERK-CHOP signaling pathway [23]. Similar results were observed after RCN1 knockdown in sorafenib-sensitive Huh7 cells after TM treatment (Fig. S3B).

**Fig. 3:**
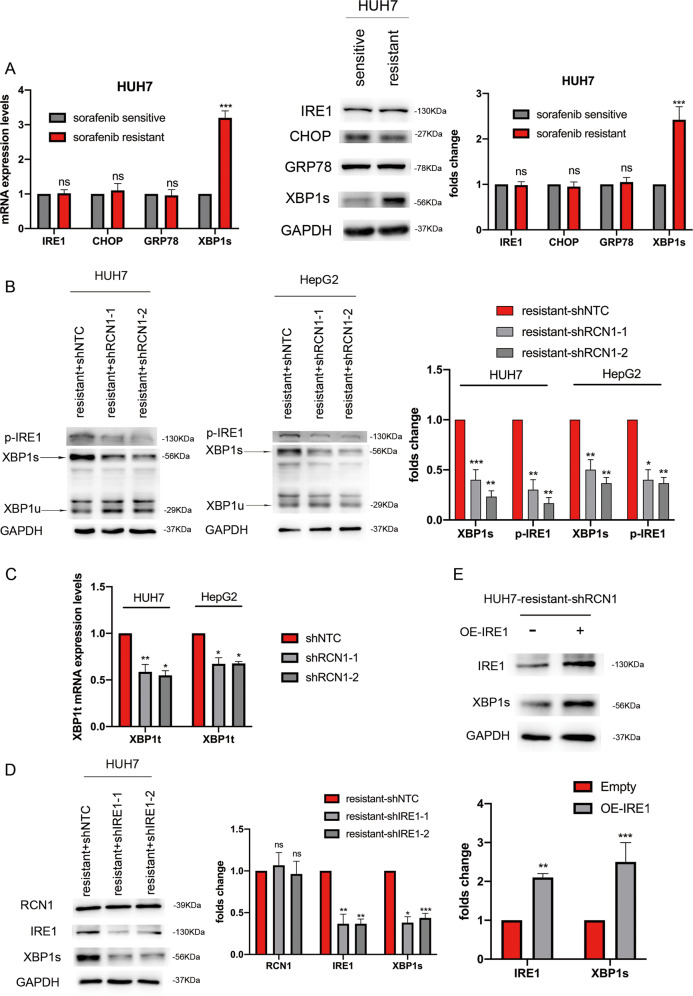
RCN1 contributes to selective activation of the unfolded protein response. **A** qRT-PCR and western blot analysis showing the mRNA and protein levels of IRE1, CHOP, GRP78, and *XBP1s* in sorafenib-resistant and sorafenib-sensitive Huh7 cells. **B** Western blot analysis showing protein levels of XBP1 and p-IRE1α in sorafenib-resistant Huh7 cells with or without RCN1 silencing. **C** Total mRNA levels of *XBP1* in RCN1-knockdown drug-resistant cells. **D**, **E** Western blot analysis of RCN1, IRE1α, and XBP1 expression in sorafenib-resistant Huh7 cells with downregulated or overexpressed IRE1. Data are presented as the means ± SEM of three independent experiments; data for western blot have undergone quantitative analysis. ns not significantly different. *P < 0.05; **P < 0.01; ***P < 0.001, t-test.

Subsequently, we explored the expression of IRE1, CHOP, GRP78, and XBP1s in sorafenib-resistant and -sensitive cells and found XBP1s to be significantly upregulated in sorafenib-resistant cells (Fig. 3A and S3C). In addition, knocking down RCN1 in sorafenib-resistant Huh7 and HepG2 cells significantly reduced the expression of p-IRE1α and XBP1s (Fig. 3B), although it had no effect on the expression of p-PERK, CHOP, and GRP78 (Fig. S3D). Moreover, the expression of p-IRE1α and XBP1s was also increased in RCN1-overexpressing sorafenib-sensitive Huh7 cells (Fig. S3E). The results suggested that in sorafenib-resistant cells, RCN1 may activate the UPR via the IRE1α–XBP1s pathway instead of inhibiting ER stress through the PERK-CHOP pathway. Notably, the total protein content of XBP1 was also significantly reduced in RCN1-knockdown drug-resistant cells (Fig. 3C).

Finally, we investigated whether IRE1α is necessary for RCN1-mediated XBP1 splicing. IRE1α silencing in sorafenib-resistant cells markedly reduced XBP1 splicing, suggesting that IRE1α is required for RCN1-mediated XBP1 splicing (Fig. 3D). To confirm this, we examined the effect of IRE1α overexpression on XBP1 splicing in RCN1-deficient cells. IRE1α overexpression upregulated XBP1 splicing in sorafenib-resistant RCN1-knockdown cells (Fig. 3E). Taken together, our results indicated that in sorafenib-resistant cells, RCN1 has a specific role in the regulation of the IRE1α–XBP1 pathway as part of the UPR.

### IRE1α–XBP1s signaling is crucial for sorafenib resistance and HCC malignancy

**Fig. 4:**
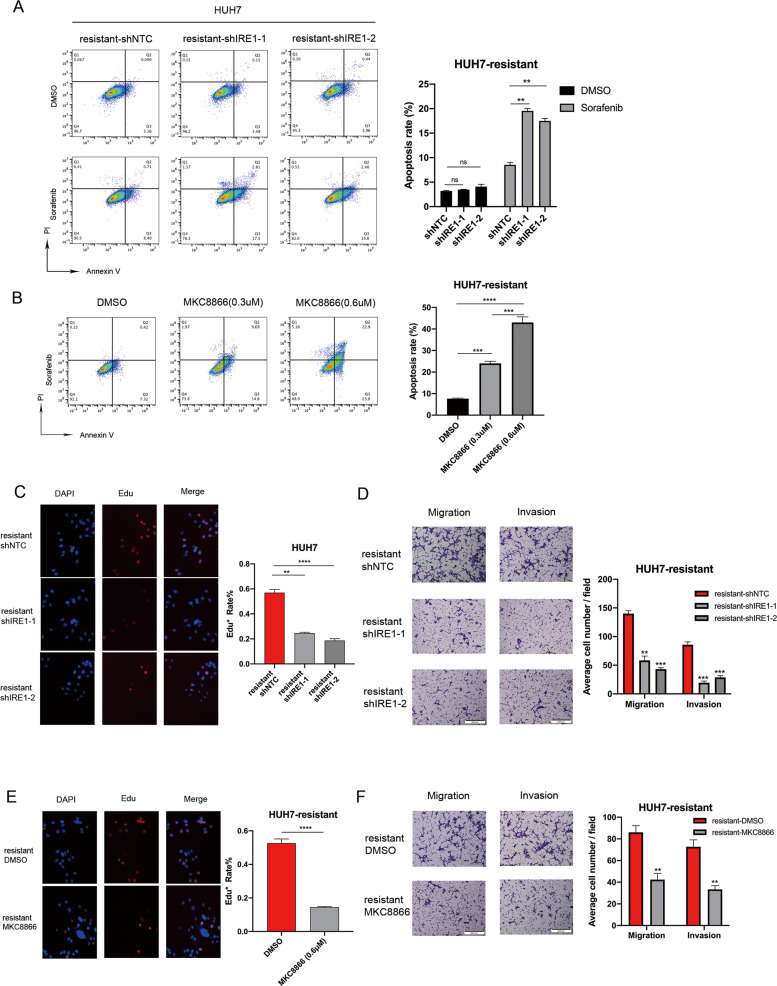
The IRE1α–XBP1s pathway is crucial for drug resistance and HCC malignancy. **A** Representative flow cytometry analysis of Annexin V-PI staining in sorafenib-resistant Huh7 cells with or without RCN1 silencing, in the presence of 5 μM sorafenib. **B** Representative flow cytometry analysis of Annexin V-PI staining in sorafenib-resistant Huh7 cells in the presence of 5 μM sorafenib and different doses of MKC8866. **C** Cell proliferation in sorafenib-resistant Huh7 cells with IRE1a knockdown assessed using an EdU assay. **D** Transwell assays of cell migration and invasion in sorafenib-resistant Huh7 cells after IRE1α knockdown. **E** Cell proliferation in sorafenib-resistant Huh7 cells assessed using EdU assays after treatment with 0.3 μM MKC8866. **F** Transwell assays of cell migration and invasion in sorafenib-resistant Huh7 cells in the presence of 0.3 μM MKC8866. Data are presented as the means ± SEM of three independent experiments. ns not significantly different. **P < 0.01; ***P < 0.001; ****P < 0.0001, *t*-test.

Depending on the duration and degree of ER stress, UPR can either provide survival signals by activating adaptive and anti-apoptotic pathways or activate signaling programs that induce cell death [24]. Therefore, we speculated that the anti-apoptotic effect of RCN1 on sorafenib-resistant cells might depend on the IRE1α–XBP1 signaling pathway. Interestingly, after knocking down IRE1α, the resistance to sorafenib could be reversed (Fig. 4A). Additionally, we used MKC8866, an optimized IRE1α RNase-specific inhibitor that can suppress XBP1s expression, to study the effect of XBP1s in sorafenib-resistant cells. MKC8866 treatment could also reverse the resistance of sorafenib-resistant cells against sorafenib-induced cell death (Fig. 4B). Therefore, we surmised that the pro-survival effect of RCN1 in drug-resistant cells depended on the IRE1α–XBP1 signaling pathway. Interestingly, IRE1α shRNA attenuated the effects of RCN1 on proliferation (Fig. 4C) and also largely eliminate the cell invasion- and migration-promoting effect of RCN1 in sorafenib-resistant cells (Fig. 4D). Moreover, MKC8866 also reduced the cell proliferation, invasion, and migration abilities of drug-resistant cells (Fig. 4E, F).

### The RCN1 EFh1/2 domain binds to GRP78 and promotes the dissociation of GRP78 from IRE1

**Fig. 5:**
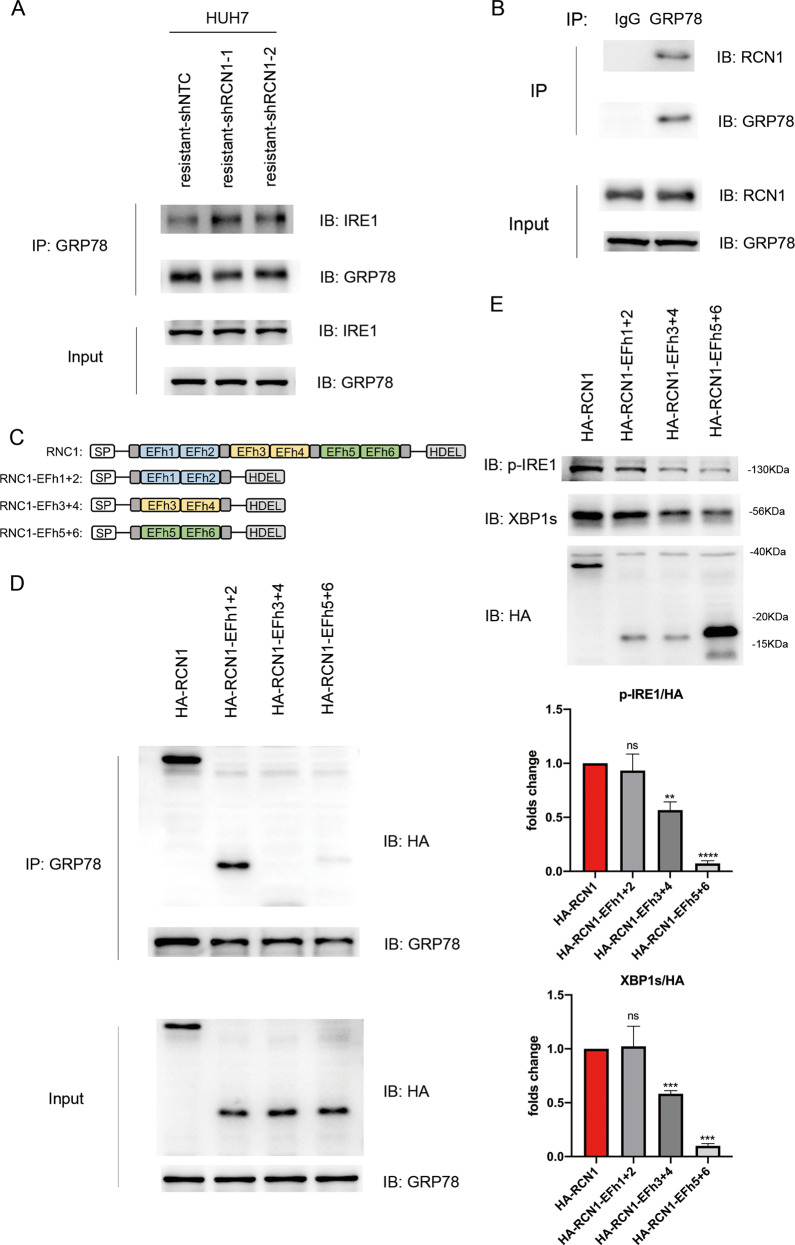
The RCN1 EFh1/2 domain binds with GRP78 and promotes the dissociation of GRP78 from IRE1. **A** Immunoblot analysis of the interaction between GRP78 and IRE1α using immunoprecipitates from sorafenib-resistant Huh7 cells with or without RCN1 silencing. **B** Immunoblot analysis of complex formation between endogenous RCN1 and GRP78 immunoprecipitated from sorafenib-resistant Huh7 cells. **C** Schematic representation of the whole-length and deletion RCN1 mutants. **D** Immunoblot analysis of complex formation between endogenous GRP78 and HA-tagged mutant RCN1. **E** Immunoblot analysis of IRE1α phosphorylation and XBP1s protein levels in sorafenib-resistant Huh7 cells transfected with whole-length and deletion RCN1 mutants. Data are presented as the means ± SEM of three independent experiments. ns not significantly different. **P < 0.01; ***P < 0.001; ****P < 0.0001, *t*-test.

According to the GEPIA database, the mRNA expression of RCN1 was not correlated with that of GRP78, IER1, and XBP1 in HCC (Fig. S4A). We aimed to ascertain whether RCN1 could influence the IRE1α-XBP1 pathway in other ways. After dissociation from GRP78, IRE1α undergoes dimerization and autophosphorylation, triggering conformational changes that activate its endoribonuclease domain, causing a reading frameshift and the translation of the active transcription factor XBP1 [25]. We examined whether the presence of RCN1 promotes the dissociation of GRP78 from IRE1α in sorafenib-resistant cells. Surprisingly, RCN1 silencing greatly increased the binding of GRP78 to IRE1α (Fig. 5A), consistent with the decrease in p-IRE1α levels observed after RCN1 knockdown (Fig. 3C). Using co-immunoprecipitation (co-IP) to examine cell lysates, we found that RCN1 and GRP78 interact physically in sorafenib-resistant Huh7 cells (Fig. 5B). To further identify the motif via which RCN1 interacts with GRP78, different HA-tagged RCN1 mutants were transfected into sorafenib-resistant cells. (Fig. 5C). Co-IP revealed that the EFh1/2 domain of RCN1 was required for interaction with GRP78 in sorafenib-resistant cells (Fig. 5D).

To investigate the effect of the GRP78–RCN1 interaction on IRE1α activation, we evaluated the levels of IRE1α phosphorylation and XBP1 splicing in sorafenib-resistant Huh7 cells (Fig. 5E). Taken together, our results suggested that RCN1 regulated IRE1α activation via its EFh1/2 domain-mediated interaction with GRP78 in sorafenib-resistant cells.

### RCN1 activates c-MYC signaling via the IRE1α–XBP1s pathway in sorafenib-resistant cells

**Fig. 6:**
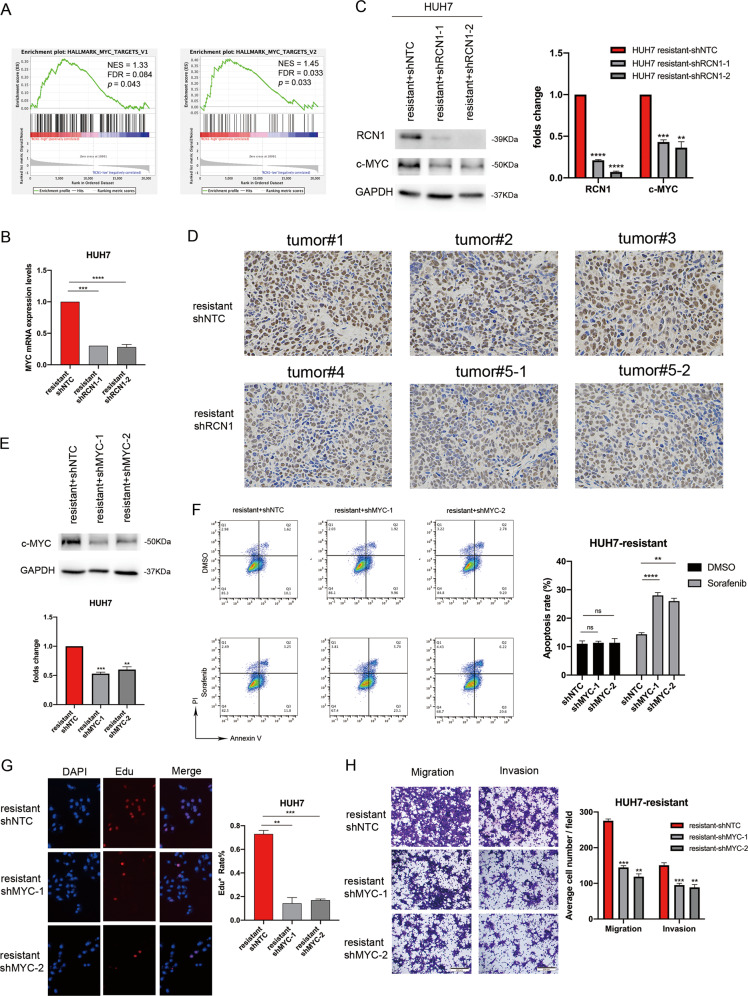
RCN1 activates c-MYC signaling in sorafenib-resistant cells. **A** Signaling pathways affected by RCN1, analyzed using GSEA based on TCGA data. **B** mRNA levels of *MYC* in RCN1-knockdown sorafenib-resistant cells. **C** Western blot analysis showing protein levels of RCN1 and c-MYC in sorafenib-resistant Huh7 cells with or without RCN1 repression. **D** Intensity of c-MYC immunohistochemical staining in xenograft tumor sections. **E** Knockdown efficiency of MYC analyzed using western blot. **F** Apoptosis of sorafenib-resistant Huh7 cells with MYC knockdown in the presence of 5 μM sorafenib analyzed using flow cytometry. **G** Cell proliferation in sorafenib-resistant Huh7 cells with MYC knockdown assessed using an EdU assays. **H** Transwell assays of cell migration and invasion in sorafenib-resistant Huh7 cells after MYC knockdown. Data are presented as the means ± SEM of three independent experiments; data for western blot have undergone quantitative analysis. ns not significantly different. **P < 0.01; ***P < 0.001; ****P < 0.0001, *t*-test.

**Fig. 7:**
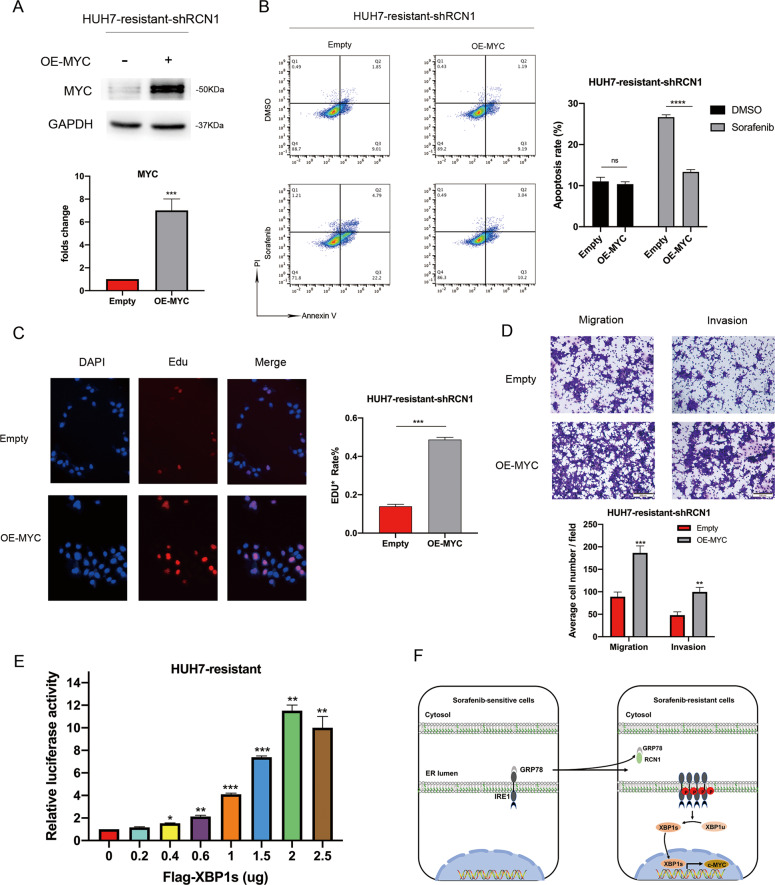
RCN1 activates c-MYC signaling through the IRE1α-XBP1s pathway in sorafenib-resistant cells. **A** c-MYC expression in sorafenib-resistant Huh7 cells with downregulated RCN1. **B** Flow cytometry analysis of Annexin V-PI staining in Huh7 RCN1-knockdown sorafenib-resistant cells with or without MYC overexpression, in the presence of 5 μM sorafenib. **C**, **D** MYC overexpression rescues the inhibitory effect of RCN1 shRNA on the. proliferation, migration, and invasion of sorafenib-resistant cells. **E** Sorafenib-resistant Huh7 cells were transfected MYC luciferase reporter plasmids. with or without a flag-XBP1s plasmid. After 24 h, the cells were harvested for a luciferase activity assay. **F** Schematic model. The increasing expression of RCN1 results in an activation of the IRE1α–XBP1s–c-MYC axis in sorafenib-resistant cells. Data are presented as the means ± SEM of three independent experiments; data for western blot have undergone quantitative analysis. ns: not significantly different. *P < 0.05; **P < 0.01; ***P < 0.001; ****P < 0.0001, *t*-test.

In order to further identify the signaling pathways regulated by RCN1, we conducted GSEA by analyzing The Cancer Genome Atlas data. We observed that the activity of the c-MYC signaling pathway was positively correlated with the expression of RCN1 in 376 HCC tissues (Fig. 6A). In addition, according to the TIMER and GEPIA databases, the expression of RCN1 was correlated with the expression of MYC in HCC (Fig. S4B, C). Moreover, c-MYC levels were significantly higher in sorafenib-resistant cells than in sorafenib-sensitive cells (Fig. S4D). We observed reduced mRNA levels of *MYC* in RCN1-knockdown sorafenib-resistant cells (Fig. 6B), and accordingly, c-MYC protein levels were also found to be lower (Fig. 6C). Interestingly, c-MYC was also upregulated in RCN1-overexpressing sorafenib-sensitive Huh7 cells (Fig. S4E). Further, the intensity of c-MYC immunohistochemical staining in xenograft tumor sections from shRCN1 mice was significantly weaker than that in sections from shNC mice (Fig. 6D). Next, we stably knocked down MYC (Fig. 6E) in sorafenib-resistant cells Huh7 cells; we found an increase in cell apoptosis (Fig. 6F) along with a reduction in cell proliferation (Fig. 6G), invasion, and migration (Fig. 6H). MYC overexpression after RCN1 knockdown (Fig. 7A) decreased the sensitivity of drug-resistant Huh7 cells to sorafenib (Fig. 7B). Importantly, MYC overexpression rescued the inhibitory effect of RCN1 shRNA on proliferation, migration, and invasion in sorafenib-resistant cells (Fig. 7C, D).

Previous studies have shown that the IRE1α–XBP1s pathway can promote the development of prostate cancer by activating c-MYC signaling [11]. Therefore, we speculated that the increased XBP1 splicing caused by RCN1 overexpression in sorafenib-resistant cells might further affect the activation of the c-MYC signaling pathway. We analyzed the mRNA levels of *MYC* in IRE1-knockdown sorafenib-resistant cells and cells treated with MKC8866 and found that, as expected, the mRNA levels of MYC were both decreased in both (Fig. S4F). We found that the ectopic expression of flag–XBP1s in sorafenib-resistant cells activated the luciferase reporter driven by the c-MYC promoter in a dose-dependent manner (Fig. 7E). Therefore, our results showed that c-MYC signaling contributed to sorafenib resistance and HCC malignancy via the RCN1–IRE1α–XBP1s pathway (Fig. 7F).

### A variety of cytokines and drugs lead to increased RCN1 expression

**Fig. 8:**
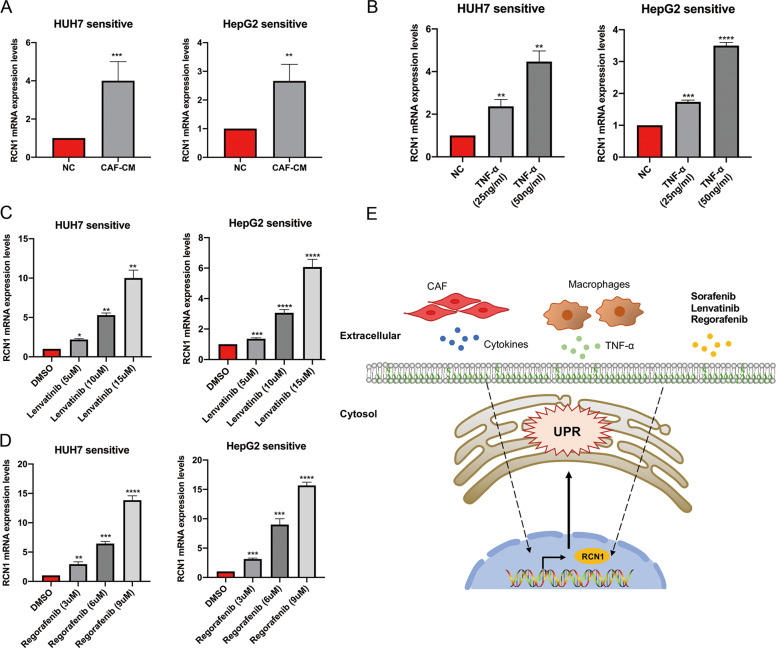
A variety of cytokines and drugs lead to an increased expression of RCN1. **A** qRT-PCR analysis showing the mRNA levels of *RCN1* in Huh7 and HepG2 cells cultured with or without CAFs-CM (NC: negative control) for 48 h. **B** qRT-PCR results showing the mRNA expression of *RCN1* in Huh7 and HepG2 cells in response to 25 ng/ml and 50 ng/ml of TNFα treatment for 24 h. **C**, **D** *RCN1* mRNA is overexpressed after increased doses of lenvatinib and. regorafenib. **E** Schematic model. The tumor microenvironment and a variety of drugs can. promote the expression of RCN1. Data are presented as the means ± SEM of three independent experiments. *P < 0.05; **P < 0.01; ***P < 0.001; ****P < 0.0001, *t*-test.

The tumor microenvironment refers to the environment surrounding a tumor. Cancer-associated fibroblasts (CAFs) are important tumor stromal cells [26]. We collected conditioned medium from patient-derived CAFs and co-cultured Huh7 or HepG2 cells in this medium in vitro. qRT-PCR showed that *RCN1* was upregulated in Huh7 and HepG2 cells (Fig. 8A). However, the specific cytokines secreted by CAFs that contributed to the increase in *RCN1* levels needed to be identified. It has been reported that the TNF-α-NFκB pathway is necessary for the increased expression of RCN1 [23]. Interestingly, TNF-α is mainly released from macrophages, and TNF-α can directly lead to tumor promotion or the apoptosis of tumor cells [27, 28]. Therefore, we added recombinant human TNF-α into the condition medium of Huh7 and HepG2 at a gradient concentration and found that *RCN1* was upregulated in these cells in a concentration-dependent manner (Fig. 8B).

Lenvatinib is an oral small-molecule inhibitor of multi-receptor tyrosine kinases that has been approved for the first-line treatment of unresectable HCC. Lenvatinib dually inhibits the VEGF and FGF pathways and blocks proliferation signals from VEGFR and FGFR, which are upregulated in HCC. Interestingly, we found that RCN1 knockdown caused a decrease in VEGFR (Fig. S4G). Additionally, RCN1 expression increased with increased doses of lenvatinib (Fig. 8C).

Regorafenib, a second-line treatment for patients with advanced HCC, has also provided some survival benefit in those showing progression on sorafenib treatment [29]. We found that RCN1 was upregulated after treatment with regorafenib in a dose-dependent manner (Fig. 8D). These results showed that the tumor microenvironment and a variety of drugs could promote RCN1 expression in liver cells (Fig. 8E).

## Discussion

HCC, one of the most common malignant tumors in the world [30], often results in a poor prognosis. Sorafenib is used as the first-line therapy for patients with advanced liver cancer. However, sorafenib responsiveness varies among patients with advanced HCC and most of them eventually develop drug resistance, necessitating the elucidation of the molecular mechanisms underlying sorafenib resistance.

In the present study, we found that RCN1 expression was upregulated in sorafenib-resistant HCC cells and that the presence of RCN1 conferred sorafenib resistance to these cells. Clinically, high RCN1 levels in human HCC were predictive of a worse prognosis, suggesting that RCN1 could serve as a biomarker in individualized HCC therapy. Mechanistic studies revealed that RCN1 blunts the efficacy of sorafenib and induces malignancy in HCC by activating the IRE1–XBP1s-c-MYC pathway. Therefore, RCN1 may be an alternative target for HCC treatment.

GO enrichment analysis of the GSE94550 dataset (Fig. S1B) revealed enrichment for widely distributed and extensive GO terms under the “biological processes” domain. High enrichment was observed for “signal transduction,” “positive regulation of cell proliferation,” “response to hypoxia,” and “steroid metabolic process,” suggesting that the biological processes of sorafenib-resistant cells are highly different. Due to the limitations of differentially expressed gene (DEG) enrichment analysis, some important genes with minor changes may have been excluded. Thus, we conducted GSEA to further explore the functional changes related to sorafenib resistance (Fig. S1D). As expected, GSEA of the HALLMARK gene set revealed a dramatic impairment in EMT. Accordingly, in our study, we found that RCN1 is closely related to EMT. The TNF-α-NFκB pathway, which plays a pivotal role in regulating cell proliferation, was significantly upregulated in sorafenib-resistant cells. Interestingly, it has been reported that RCN1 can be upregulated via the TNF-α-NFκB pathway [23]. Moreover, the HYPOXIA gene set was also upregulated in sorafenib-resistant cells, consistent with the DEG enrichment results. Compared with normal liver tissue, liver cancer tissue has abnormally low perfusion, which leads to severe hypoxia. In addition, the anti-vascular effects of sorafenib aggravate hypoxia. Three main O_2_-sensing pathways promote hypoxia tolerance [31]. First, hypoxia stabilizes hypoxia-inducible factor (HIF) 1α, which facilitates the transcriptional activation of several genes. Second, the activity of mTORC1, an important integrator of metabolic signals, is inhibited by hypoxia. Third, hypoxia can trigger the UPR. In recent years, evidence from both laboratory and clinical studies has indicated that hypoxia is a strong activator of the UPR. XBP1, an essential survival factor, is necessary under hypoxic conditions [32]. XBP1 maintains the hypoxia response by regulating HIF1α transcription, thereby ensuring maximum HIF activity and an adaptive response to the cytotoxic microenvironment of tumors [9]. It has been suggested that RCN1 promotes cancer cell survival under hypoxia [33]. Additional research is required to explore the relationship between RCN1 expression and hypoxia.

There have been reports that RCN1 is aberrantly expressed in a variety of tumors. However, there are limited studies on the role of RCN1 in tumors, and the role of RCN1 in the development and treatment of liver cancer is still unknown. In the present study, we report for the first time the vital role of RCN1 and its downstream pathway in sorafenib resistance in HCC. It is known that RCN1 inhibits PERK-CHOP-mediated UPR signaling during ER stress to prevent TM-induced cell death [14, 21, 23]. Surprisingly, however, we found that in sorafenib-resistant cells, RCN1 activates the IRE1α–XBP1 pathway to constitutively activate the UPR and thereby reduces sorafenib-induced apoptosis. We speculate that this may be one reason for sorafenib resistance in Huh7 and HepG2 cells. In response to chronic stress, many cancer cells activate the UPR through the IRE1α–XBP1 pathway in order to survive. IRE1α has been implicated in tumor tolerance against hypoxia and angiogenesis, and XBP1s, a transcription factor previously shown to be associated with cancer, is also elevated in hypoxic cells, where it provides a survival advantage [34]. In prostate cancer cells, Bag5 overexpression inhibits ER stress-induced apoptosis in the UPR by suppressing PERK-eIF2-ATF4 activity while enhancing the IRE1α–XBP1 axis [35]. Tay et al. demonstrated that sustained IRE1 signaling in the UPR is an important protective mechanism against ER stress-induced apoptosis in melanoma cells [36]. In particular, when HCC cells encounter ER stress due to in vitro sorafenib treatment, the activation of the IRE1α–XBP1 signaling pathway plays a protective role [8]. Our study proposes for the first time that RCN1 can activate the UPR under certain circumstances and promote tumor cell survival instead of inhibiting PERK-CHOP-mediated UPR signaling during ER stress.

In cancer, the genetic deregulation of MYC expression drives malignant transformation [37] and is implicated in hepatocarcinogenesis [38]. The activation of c-MYC can promote the resistance of liver cancer cells to sorafenib [13, 39]. Previous research has established a link between UPR and c-MYC, and it is known that the IRE1α–XBP1s pathway promotes prostate cancer development by activating c-MYC signaling. Here we demonstrate, for the first time, that the central oncogenic c-MYC signaling pathway, previously shown to have important roles in HCC, is directly activated by IRE1α signaling via XBP1s in sorafenib-resistant cells. Interestingly, we found that the total levels of XBP1 were also decreased in RCN1-knockdown sorafenib-resistant cells (Fig. 3C). Hong et al. [40] found that MYC activates the pro-survival IRE1α–XBP1 pathway in HCC. Additional studies are required to establish whether c-MYC regulates IRE1α expression and XBP1 activity in sorafenib-resistant cells. If so, this would imply that the XBP1s-c-MYC axis enables a powerful feedback loop to establish a survival pathway in sorafenib-resistant cells.

In this study, we demonstrated that RCN1 is overexpressed in sorafenib-resistant cells and activates the IRE1α–XBP1s–c-MYC pathway. Moreover, RCN1 is upregulated in the presence of lenvatinib, regorafenib, and certain cytokines. Our results reveal the role of the IRE1α–XBP1s–c-MYC axis in sorafenib-resistant HCC cells and the molecular mechanisms of its action. These findings suggest that targeting RCN1 may help in preventing sorafenib resistance in HCC and inhibit tumor progression. In the future, we will explore the role of RCN1 in the HIF1α signaling pathway and evaluate whether c-MYC regulates IRE1α expression and XBP1 activity in sorafenib-resistant cells. Additional studies will also be necessary to investigate the role of RCN1 in tumor stromal cells.

## Materials and methods

### Cell culture

The human HCC cell lines (MHCC-LM3, MHCC-97H, Hep3B, HepG2, and Huh7) were procured from the Shanghai Institutes for Biological Sciences (China) Cell Center. In order to establish sorafenib resistance in vitro, these cells were exposed to 1.5 μM sorafenib at first, and this concentration was gradually increased to 6 μM via weekly increases for 8 months. All cell lines were incubated in humidified air containing 5% CO_2_ at 37 °C and cultured in Dulbecco’s Modified Eagle’s Medium (DMEM) containing 10% fetal bovine serum (FBS), 100 IU/mL penicillin, and 100 μg/mL streptomycin.

### Immunohistochemistry

For immunohistochemistry (IHC), we prepared formalin-fixed paraffin-embedded primary HCC tissue sections and resected xenografts according to the manufacturer’s protocol (Servicebio, GP1001, GP1010) and incubated them with the corresponding primary antibodies at 37 °C for 2 h. Following this, they were incubated with goat anti-rabbit antibody against immunoglobulin G (Servicebio, G1213). The primary IHC antibodies were as follows: anti-RCN1 (Abcam, ab210404), anti-PCNA (Servicebio, GB11010), and anti-c-MYC (Proteintech, 10828-1-AP).

### Animal experiments

All experimental protocols and animal care methods were approved by the guidelines of the Nanjing Medical University (NJMU) Institutional Animal Care and Use Committee. In order to evaluate the tumor-formation capacity of the sorafenib-resistant cells, ~1 × 10^5^ cells with or without RCN1 knockdown were suspended in 100 μl phosphate-buffered saline (PBS) and then transplanted subcutaneously into the flank of 4–6-week-old male NOD/SCID mice (Vital River, Beijing, China) (randomly divided into different groups, with 10 mice per control group and 10 mice per experimental group). The mice were maintained under specific pathogen-free conditions. Tumor development was monitored regularly. Tumor volume was calculated using the following formula: *V* = (*L* × *W*^2^)/2 (*V*, volume; *L*, length diameter; *W*, width diameter). When the tumors reached 5 mm × 5 mm (*L* × *W*) in size, the mice were randomized for treatment with sorafenib (25 mg/kg/day, orally) or vehicle (DMSO). After 3–4 weeks, mice were killed, and the tumors were dissected.

### Vector construction

For the knockdown of gene expression, short-hairpin RNAs (shRNAs) were designed and cloned into the lentiviral vector pLKO.1-puro. The empty vector was used as a negative control. Puromycin (2 µg/mL) (Thermo Fisher Scientific, USA) was used to generate antibiotic-resistant cells for subsequent assays. The sequences of the shRNAs are shown in Supplementary Table S1. RCN1 EF-hand deletion mutants were constructed using pcDNA3.1-HA vectors. The cloning primers are shown in Supplementary Table S2. *IRE1*, *MYC*, and *XBP1s* cDNA was also inserted into pcDNA3.1 or pcDNA3.1-Flag vectors, and the empty vector was used as a negative control.

### RNA extraction and quantitative real-time PCR

RNA was extracted from harvested cells using the TRIzol reagent (Invitrogen, USA). The total RNA (1 µg) was reverse transcribed into cDNA according to the manufacturer’s instructions (Vazyme, R323-01). An equivalent volume of cDNA per sample was prepared for real-time PCR analysis using SYBR Green (Vazyme, Q711-02). qRT-PCR data were analyzed using the relative gene expression method and normalized based on *GAPDH* levels. The primer sequences used for qRT-PCR are shown in Supplementary Table S3.

### Statistical analysis

All data were analyzed using GraphPad Prism 8.0 software (La Jolla, CA, USA) and SPSS 24.0. All testing was done blind, in duplicate by two technicians. Statistical significance was determined by a two-tailed unpaired *t*-tests (2 groups), or one-way ANOVA (>2 groups) with Bonferroni’s multiple comparisons test. All in vitro functional assays are representations of at least three independent experiments expressed as mean ± SD. *P*-values < 0.05 were considered statistically significant.

Additional experimental procedures are provided in the Supplementary Information.

## Supplementary information

Supplemental Material

Authorship Addition Justification Approval Form

Attribution of Authorship

## Data Availability

The datasets generated and/or analyzed during the current study are available from the corresponding authors on reasonable request.
