# Supplementary material for: RCN1 induces sorafenib resistance and malignancy in hepatocellular carcinoma by activating c-MYC signaling via the IRE1α–XBP1s pathway
Source: Cell Death Discov. 2021 Oct 18;7:298. doi: 10.1038/s41420-021-00696-6 (PMC8523720; doi:10.1038/s41420-021-00696-6)
Supplement: Supplementary file 1 — Supplemental Material [file 41420_2021_696_MOESM1_ESM.docx]

**Supplementary Information For**

**RCN1 induces sorafenib resistance and malignancy in hepatocellular carcinoma by activating c-MYC signaling via the IRE1α-XBP1s pathway**

**Jia-Wei Wang · Li Ma · Yuan Liang · Xiao-Jun Yang · Song Wei · Hao Peng · Shi-Pei Qiu · Xu Lu · Ya-Qing Zhu & Bao-Lin Wang**

Correspondence to:

Ya-Qing Zhu (doctorzhuyq@163.com) and Bao-Lin Wang (wangbl@njmu.edu.cn)

This file includes:

Supplementary Methods

Supplementary Tables S1 to S3

Supplementary Figures S1 and S4

**Supplementary Methods**

**Patients and clinical samples**

Sixty-eight HCC tissue samples were obtained from patients who underwent hepatectomy at the Department of Hepatobiliary Surgery, Nanjing Medical University’s First Affiliated Hospital (China) between 2017 and 2019 after they provided signed informed consent (No. 2019-SR-127). The criteria used to identify patients with HCC were based on histopathological analysis. The efficacy of sorafenib in patients was assessed according to RECIST (Response Evaluation Criteria In Solid Tumors) (RONOT M, et al. Oncologist, 2014, 19(4): 394-402). In brief, patients underwent a baseline evaluation; during this evaluation, the maximum diameters of the measurable lesions from the most recent imaging examination (within 6 months) before sorafenib treatment were recorded. Then, after sorafenib initiation, patients were scheduled to undergo an imaging examination every four weeks. If all lesions disappeared or the baseline diameter of the largest lesion reduced by more than 30%, patients were considered to be sensitive to sorafenib. Patients showing increases in lesion size, new lesions, or <30% reduction in the baseline diameter of the largest lesion were considered to be sorafenib-resistant. All procedures involving human samples were approved by the Ethics Committee of the Affiliated Hospital of Nanjing Medical University. The correlation between RCN1 expression and the clinicopathological features of all HCC patients (n=68) are shown in Table 2. In addition, the clinical characteristics of sorafenib-resistant patients (26 patients were excluded from the overall cohort of HCC patients owing to a loss to follow-up or lack of information on sorafenib treatment) are summarized in Table 1.

**Spheroid formation assay**

Approximately 150 dissociated cells were seeded in each well of an ultra-low 24-well plate (Corning Integrated Life Sciences, Acton, MA, USA) and grown in DMEM/F12 medium (Invitrogen) supplemented with 1% methylcellulose (Sigma-Aldrich, St Louis, MO, Cat. No. M7027), 20 ng/mL epidermal growth factor (Invitrogen, Cat. No. PHG0311), 20 ng/mL primary fibroblast growth factor (Invitrogen, Cat. No. PHG0266), and 1× B27 (Invitrogen, Cat. No. 12587010) in humidified air containing 5% CO_2_ at 37 °C. The spheres were counted under a stereomicroscope (Olympus, Tokyo, Japan) after 7 days.

**Western blot**

Cellular proteins were extracted from harvested cells using RIPA lysis buffer (Beyotime, China) containing phenylmethylsulfonyl fluoride (PMSF) (Beyotime, China). A Bicinchoninic acid (BCA) assay protein kit (Beyotime, China) was used to detect protein concentration. Equal amounts of proteins were subjected to 10% SDS-PAGE and transferred to polyvinylidene fluoride (PVDF) membranes (Millipore, Billerica, CA, USA). Primary antibodies were as follows: anti-RCN1(ABcam, ab210404), anti-XBP1 (ABcam, ab220783), anti-BCL-2 (CST, 15071), anti-Mcl-1 (CST, 94296), anti-E-Cadherin (CST, 3195), anti-Vimentin (CST, 5741), anti-CHOP (CST, 2895), anti-N-Cadherin (Proteintech, 22018-1-AP), anti-GRP78 (Proteintech, 11587-1-AP), anti-IRE1α (Proteintech, 27528-1-AP), anti-c-MYC (Proteintech, 10828-1-AP), GAPDH (Proteintech, 60004-1-Ig), anti-p-IRE1α (ABclonal, AP0878), anti-p-PERK (ABclonal, AP0886), anti-cleaved caspase-3 (ABmart, MB0711), and anti-HA-Tag (ABmart, M20021).

**Co-IP assay**

Non-transfected cells or cells transiently transfected with individual constructs were lysed using immunoprecipitation lysis buffer (Beyotime, P0013). The lysate was precleared with protein A/G agarose (Smart-lifesciences, SA032005) at 4°C for 30 min and incubated with anti-GRP78 (Proteintech,1:50) or normal rabbit IgG antibody (Servicebio, 1:100) at 4°C overnight and then with protein A/G agarose at 4°C for 4 h. The agarose was washed thrice with lysis buffer and boiled in 1× sample buffer. Boiled samples were subjected to immunoblot analysis.

**EdU assay**

A Cell Light EdU DNA Imaging Kit (RiboBio, Guangzhou, China) was used to detect cell proliferation according to the manufacturer’s protocol. Cells were seeded into 96-well plates at a density of 5×10^3^ cells/well. After 48 h, 100 µl EdU (50 µM) was added to each well and incubated for 2 h. Then, the cells were fixed with 4% paraformaldehyde for 30 min and permeabilized with 0.5% Triton X-100 for 10 min. Subsequently, EdU staining was performed using an Apollo567 dyeing reaction for 30 min. Finally, the DNA was stained with Hoechst 33342 for 30 min, and the proportion of nucleated cells positive for EdU was counted under a fluorescence microscope (Olympus, Tokyo, Japan). Each sample had ﬁve duplicates, and all experiments were independently repeated thrice.

**Luciferase reporter assay**

The promoter of the *c-MYC* gene was cloned into pGL3 plasmids. Then, the pGL3-MYC luciferase reporter plasmid and the pcDNA3-Flag-XBP1s plasmid were transfected into sorafenib-resistant cells with the Lipofectamine 3000 reagent (Invitrogen, Catalog Number: L3000015). Luciferase activity was determined using a luciferase assay system (Promega, E1910), and fluorescence was detected using a GloMax® 20/20 Luminometer.

**Electron microscopy**

Sorafenib-resistant cells with or without RCN1 repression were cultured at a density of 5 × 10^5^ cells/ml. Cells were pelleted through centrifugation at 1000g for 5 min and washed with PBS once before fixation. After overnight fixation, cells were sent to the Analysis and Testing Center of Nanjing Medical University for analysis. At least 50 cells from each group were analyzed, and representative images are shown.

**Annexin V-PI assay**

For detecting apoptosis, cells were stained with 5 μl FITC-conjugated Annexin V (Vazyme) and 5 μL PI (Vazyme) in 1× Annexin V binding buffer (BD Biosciences). Sorafenib was purchased from Selleck (S7397) (China). The concentration of sorafenib used for all in vitro apoptosis assays was 5 μM, and treatment was performed for 48 h. These values were in line with the IC_10_ of sorafenib-resistant Huh7 cells. The cells were processed for flow analysis using the Beckman Cytoflex according to the manufacturer’s instructions.

**Isolation of cancer-associated fibroblasts (CAFs)and normal fibroblasts (NFs)**

Fresh HCC samples from patients were cut into pieces approximately 1×1 mm in size and cultured in Dulbecco’s Modified Eagle Medium (DMEM) in the presence of 1 mg/mL collagenase I and 100 U/mL hyaluronidase at 37°C for 2 h. They were then washed twice with phosphate-buffered saline (PBS) and centrifuged at 500 x*g* for 10 min, following which the supernatants were discarded. The samples were finally resuspended in DMEM with 10% fetal bovine serum (FBS), 100 mg/mL streptomycin, and 100 IU/mL penicillin and then cultured in a 5% CO_2_ environment at 37°C. The culture medium was replaced every 2 days. All experiments were performed using fibroblasts until passage 10 to avoid CAF senescence.

**Bioinformatics analysis**

Sorafenib resistance-related gene expression datasets (GSE94550) were obtained from the Gene Expression Omnibus database (https://www.ncbi.nlm.nih.gov/ geo/). The limma package of R language (http://master.bioconductor.org/packages/release/bioc/html/limma.html) was used to perform standardized pre-processing and to screen differentially expressed genes. Differential gene expression was considered significant if it simultaneously met the requirements of a false discovery rate (FDR) ≤ 0.01 and an absolute fold change ≥ 2. Then, heatmaps were created to depict the differentially expressed genes using the pheatmap package(https://cran.r-project.org/web/packages/pheatmap/index.html). The Database for Annotation, Visualization, and Integrated Discovery (DAVID) (https://david.ncifcrf.gov/) (Huang da, Sherman & Lempicki, 2009b; Huang da, Sherman & Lempicki, 2009a) was utilized to perform Gene Ontology (GO) enrichment analysis in order to identify functional categories of DEGs. DEGs were uploaded, and the function charts were generated according to the instructions of the DAVID manual. The groups meeting the requirements of a P-value <0.05 and gene counts more than two were examined. In our study, Gene Set Enrichment Analysis (GSEA) was performed using the Java GSEA implementation (<http://software.broadinstitute.org/gsea/index.jsp>) (Subramanian et al. Proc Natl Acad Sci U S A, 2005, 102(43): 15545-50). We adopted the gene lists of KEGG or hallmark gene signature from The Molecular Signatures Database (MSigDB). The absolute signal-to-noise value of gene expression was used as a metric for ranking genes in the GSEA, but the rest of the parameters were set to default values. The GEPIA database (http://gepia.cancer-pku.cn/) and TIMER websites were used to conduct survival analyses based on RCN1 expression and to explore the correlation between the expression of RCN1 and that of other genes.

**Supplementary Tables**

**Supplementary Table S1. shRNA sequences for gene silencing.**

| Gene | Species |  | shRNA sequences |
| --- | --- | --- | --- |
| *RCN1* | Human | #1 | AGAAGCTAACTAAAGAGGAAA |
|  |  | #2 | CCGCAGAGTTTCATGATTCTT |
| *IRE1* | Human | #1 | CCCATCAACCTCTCTTCTGTA |
|  |  | #2 | CTACTGGATAAACTTGCTTCA |
| *MYC* | Human | #1  #2 | CAGTTGAAACACAAACTTGAA  CAGGAACTATGACCTCGACTA |

**Supplementary Table S2. Primer sequences for cloning the RCN1 EF-hand deletion mutants using the pcDNA3.1-HA vector.**

| RCN1 domains | Forward (5' -> 3') | Reverse (5' -> 3') |
| --- | --- | --- |
| *EFh1+2*  *(1–160 aa)* | CCGCTCGAGGCGCGCGGTGGCCGCGGCCG | ACGAGATCTTAGACTTCTTAGTACTTTGA |
| *EFh3+4*  *(161–240 aa)* | CCGCTCGAGCATCACACCTTTAAAAAGAT | ACGAGATCTTCCCGGTAAGAGGAGTACCC |
| *EFh5+6*  *(241–330 aa)* | CCGCTCGAGGAGCCAGACTGGGTTTTATC | ACGAGATCTAGTTTCGAGTAGTACTAAAA |
|  |  |  |

**Supplementary Table S3. Primer sequences for real-time PCR.**

| Gene | species | Forward (5' -> 3') | Reverse (5' -> 3') |
| --- | --- | --- | --- |
| *RCN1* | Human | AAACGGGTGCAGAAAAGATACA | AGGTAGTAACCATAGGTGGCTT |
| *XBP1t* | Human | GGCATCCTGGCTTGCCTCCA | GCCCCCTCAGCAGGTGTTCC |
| *XBP1s* | Human | CTGAGTCCGCAGCAGGTG | TCCAAGTTGTCCAGAATGCC |
| *MYC* | Human | GCTGCTTAGACGCTGGATTT | CTCCTCCTCGTCGCAGTAGA |
| *VEGFA* | Human | AGGGCAGAATCATCACGAAGT | AGGGTCTCGATTGGATGGCA |
| *CHOP* | Human | GGAAACAGAGTGGTCATTCCC | CTGCTTGAGCCGTTCATTCTC |
| *IRE1* | Human | CACAGTGACGCTTCCTGAAAC | GCCATCATTAGGATCTGGGGAGA |
| *GRP78* | Human | CATCACGCCGTCCTATGTCG | CGTCAAAGACCGTGTTCTCG |
| *GAPDH* | Human | ACAACTTTGGTATCGTGGAAGG | GCCATCACGCCACAGTTTC |

**Supplementary Figures**

**
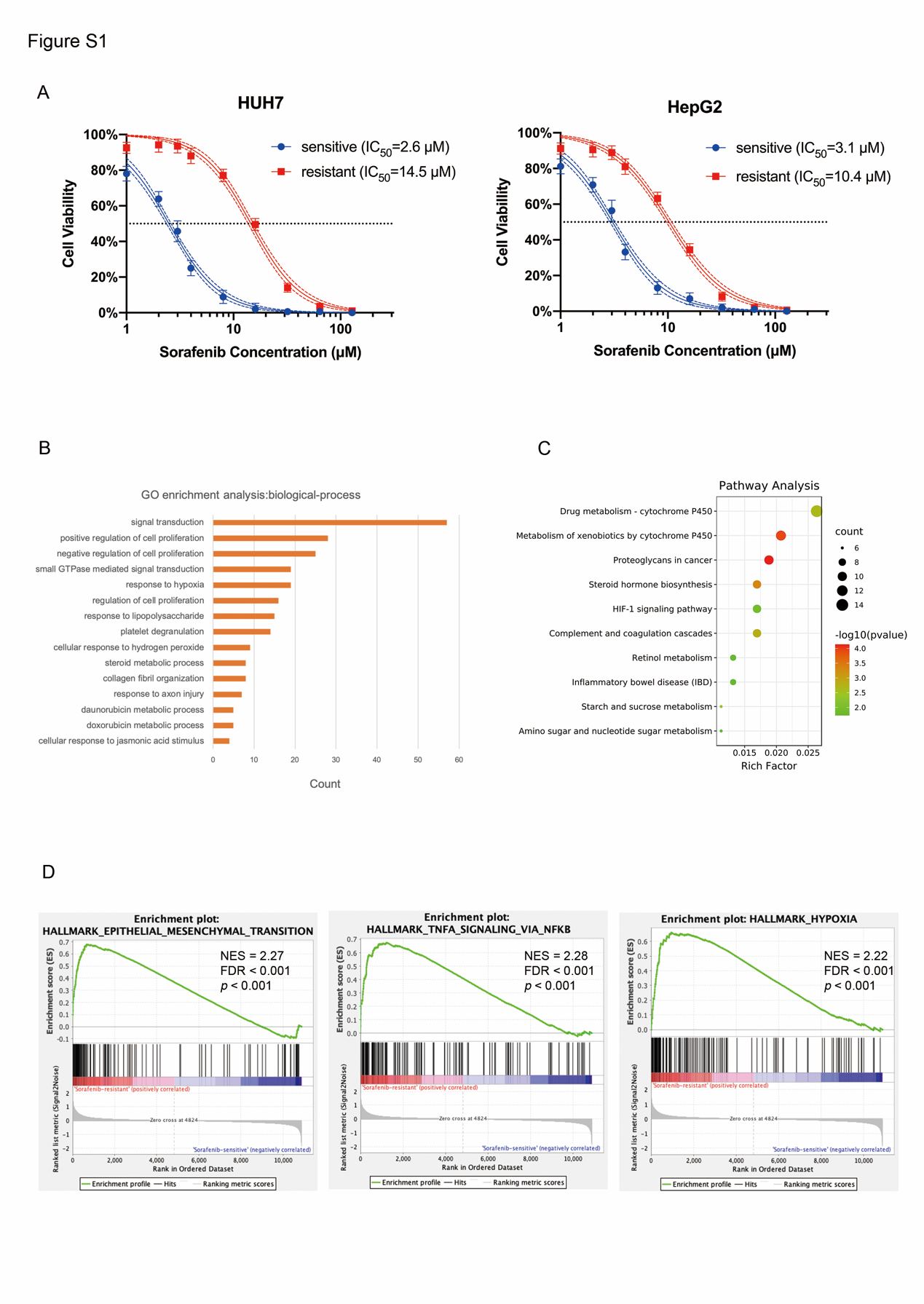
**

**Figure S1.**

1. Dose–response curves of sorafenib-sensitive or -resistant HCC cell lines at different doses of sorafenib treatment for 48 h.
2. GO enrichment analysis of the 547 upregulated genes.
3. KEGG pathways analysis of the 547 upregulated genes.
4. GSEA of the GSE94550 dataset.

**
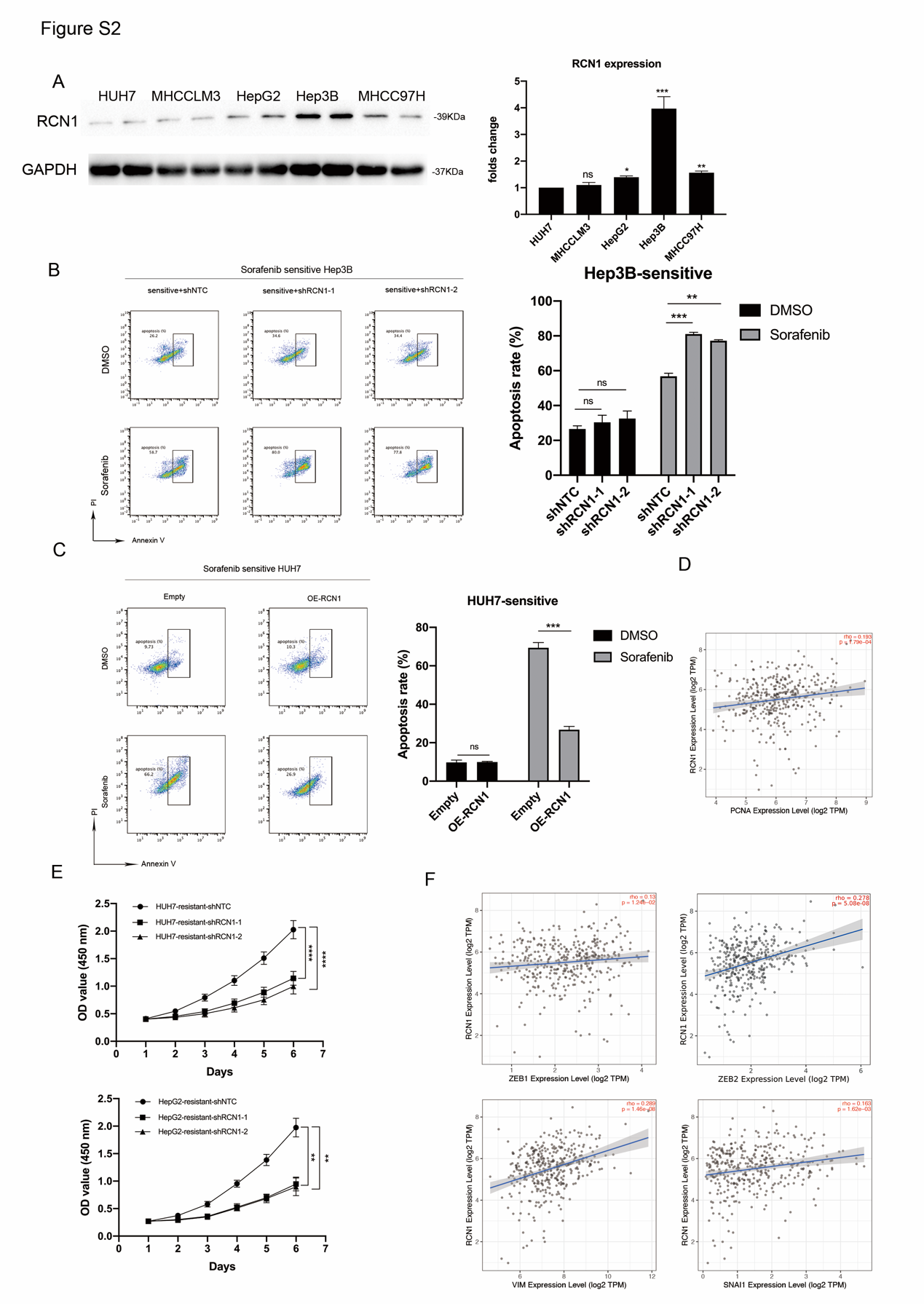
**

**Figure S2.**

1. Western blot analysis showing the protein levels of RCN1 in five HCC cell lines.
2. Representative flow cytometry analysis of Annexin V-PI staining in Hep3B sorafenib-sensitive cells with or without RCN1 silencing, in the presence of 5 μM sorafenib treatment for 48 h.
3. Apoptosis of sorafenib-sensitive Huh7 cells with MYC overexpression in the presence of 5 μM sorafenib analyzed using flow cytometry.
4. Correlation between RCN1 and PCNA expression examined using the TIMER2.0 website.
5. Cell vitality assessed using the CCK-8 assay.
6. The correlation between the expression of. EMT markers and that of RCN1 examined using the TIMER website.

Data are presented as the means ± SEM of 3 independent experiments. ns: not significantly different. *, P<0.05; **, P<0.01; ***, P<0.001; ****, P< 0.0001, t-test.

**
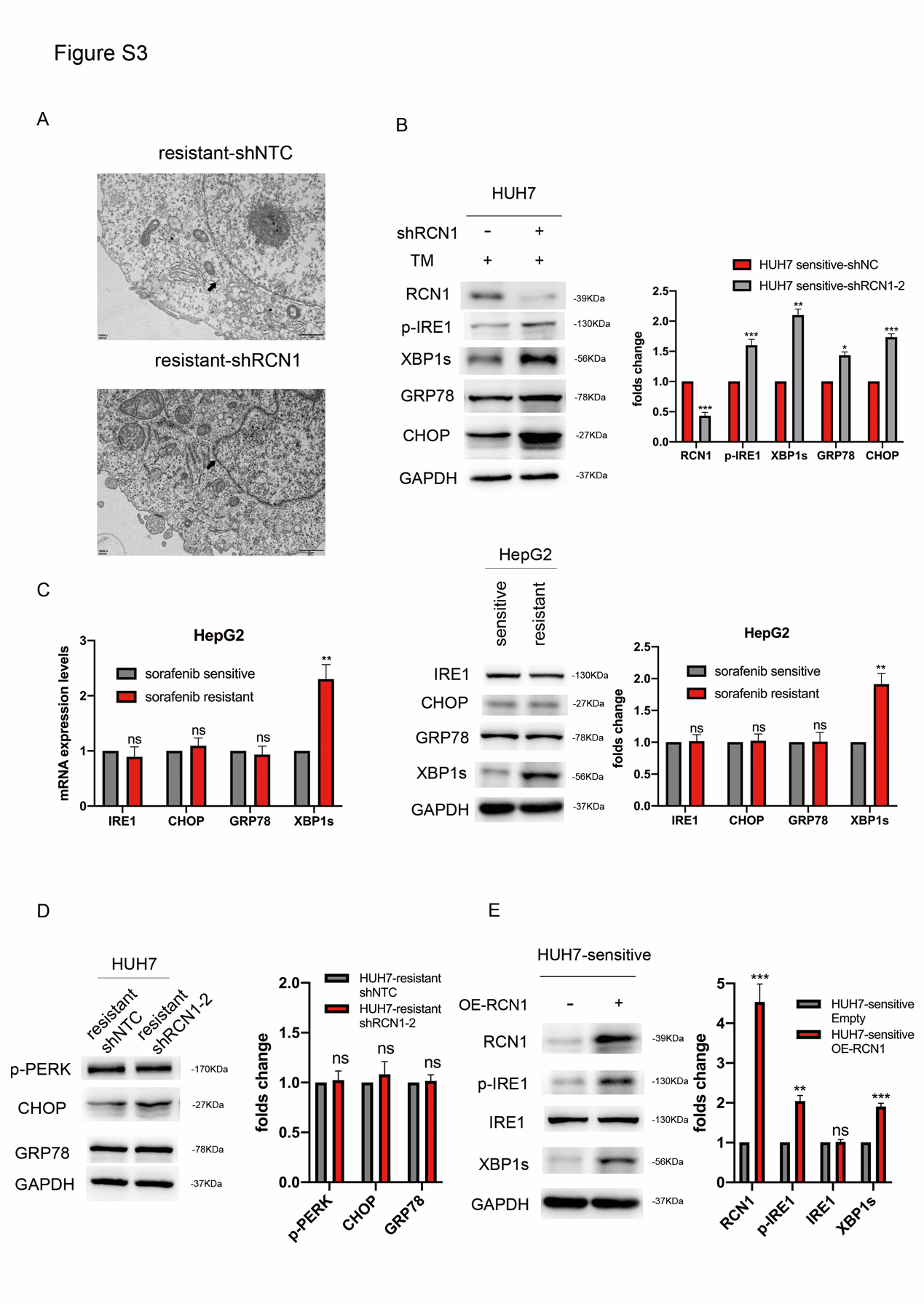
**

**Figure S3.**

1. Comparison of ER structures using transmission electron microscopy in sorafenib-resistant cells with or without RCN1 knockdown.
2. Western blot analysis showing protein levels of RCN1, p-IRE1α, XBP1s, GRP78, CHOP in Huh7 sorafenib-sensitive cells with or without RCN1 repression, in the presence of TM.
3. mRNA and protein levels of IRE1, CHOP, GRP78, and *XBP1s* in sorafenib-resistant and sorafenib-sensitive HepG2 cells.
4. Protein levels of p-PERK, CHOP and GRP78 in sorafenib-resistant Huh7 cells with or without RCN1 repression.
5. Protein levels of RCN1, p-IRE1, IRE1 and XBP1s in Huh7 sorafenib-sensitive cells with or without RCN1 overexpression.

Data are presented as the means ± SEM of 3 independent experiments. ns: not significantly different. *, P<0.05; **, P<0.01; ***, P<0.001; ****, P< 0.0001, t-test.

**
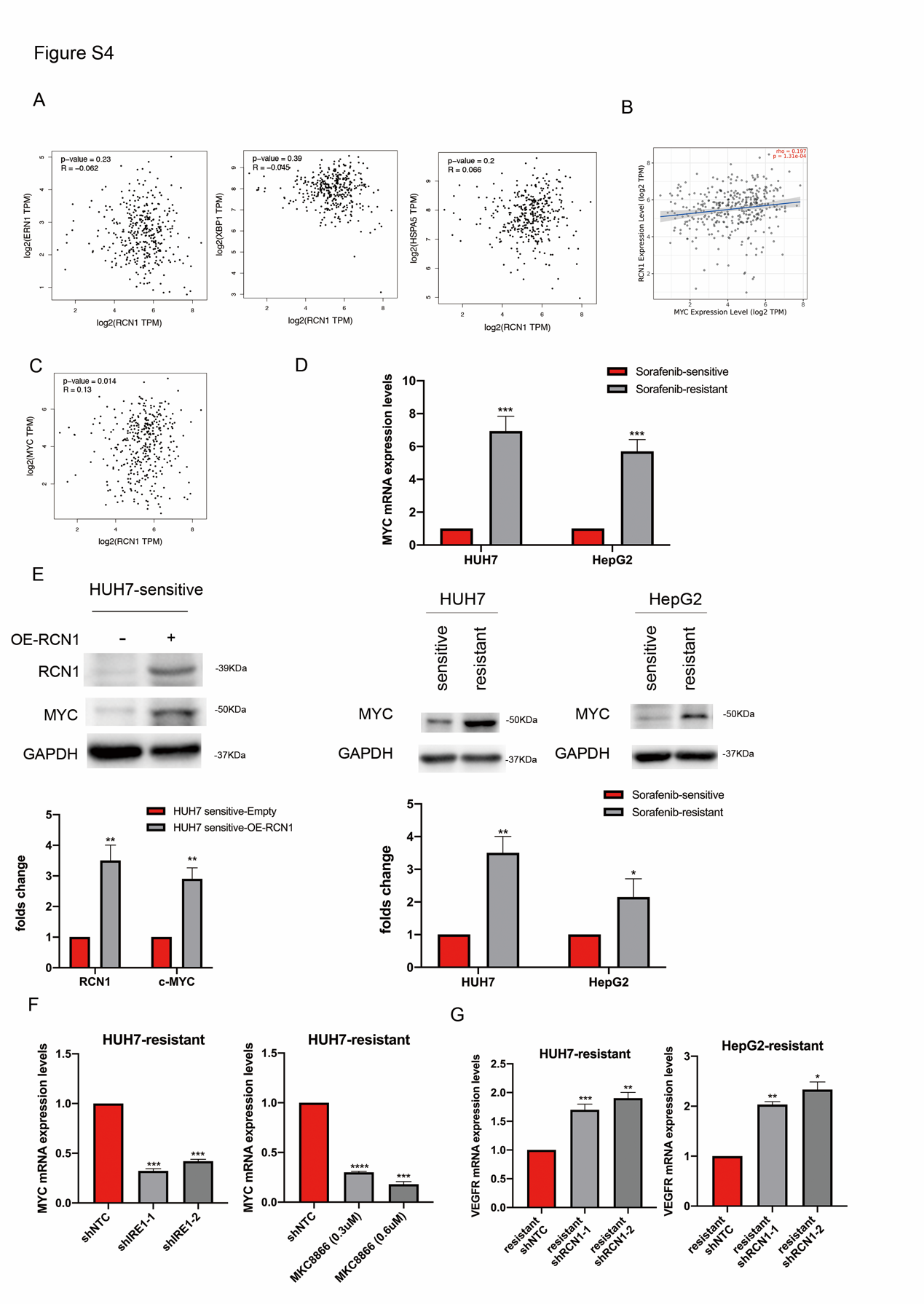
**

**Figure S4.**

1. According to the GEPIA database, the mRNA expression of RCN1 was not correlated with that of GRP78, IER1, and XBP1 in HCC.
2. Correlation between RCN1 and MYC expression examined using the TIMER2.0 website.
3. Correlation between RCN1 and MYC based on the GEPIA database.
4. Comparison of the mRNA and protein levels of MYC between sorafenib-resistant and sorafenib-sensitive cells.
5. Protein levels of MYC in Huh7 sorafenib sensitive cells with or without RCN1 overexpression.
6. mRNA level of MYC in IRE1-knockdown sorafenib-resistant cells and cells treated with MKC8866.
7. mRNA level of VEGFR in RCN1-knockdown sorafenib-resistant cells.

Data are presented as the means ± SEM of 3 independent experiments. ns: not significantly different. *, P<0.05; **, P<0.01; ***, P<0.001; ****, P< 0.0001, t-test.
