# Supplementary material for: RCN1 induces sorafenib resistance and malignancy in hepatocellular carcinoma by activating c-MYC signaling via the IRE1α–XBP1s pathway
Source: Cell Death Discov. 2021 Oct 18;7:298. doi: 10.1038/s41420-021-00696-6 (PMC8523720; doi:10.1038/s41420-021-00696-6)
Supplement: Supplementary file 2 — Authorship Addition Justification Approval Form [file 41420_2021_696_MOESM2_ESM.pdf]

SPRINGER NATURE

Change of authorship request form - Journals (pre-acceptance)

Important information. Please read.

- This form should be used by authors to request any change in authorship (adding/deleting authors) including changes in corresponding authors. This form should not be used for name changes. Please fully complete all sections. Use black ink and block capitals and provide each author's full name with the given name first followed by the family name.
- By signing this declaration, all authors guarantee that the order of the authors are in accordance with their scientific contribution, if applicable as different conventions apply per discipline, and that only authors have been added who made a meaningful contribution to the work.
- Please note, in author collaborations where there is formal agreement for representing the collaboration, it is sufficient for the representative or legal guarantor (usually the corresponding author) to complete and sign the Authorship Change Form on behalf of all authors, **next to the added/removed author(s)**. **(Complete Section 3, followed by Section 6.)**
  In author collaborations where there is no formal agreement for representing the collaboration and **there are more than 10 authors**, one may sign for all, provided the signer appends correspondence that attests that each of the authors have agreed to the change **and the added/removed authors sign the form**. **(Complete Section 3, followed by Section 6.)**
- Please note, we cannot investigate or mediate any authorship disputes. If you are unable to obtain agreement from all authors (including those who you wish to be removed) you must refer the matter to your institution(s) for investigation. Please inform us if you need to do this.
- If you are not able to return a fully completed form within **30 days** of the date that it was sent to the author requesting the change, we may have to withdraw your manuscript. We cannot publish manuscripts where authorship has not been agreed by all authors (including those who have been removed).
- Incomplete forms will be rejected.
- Please return/upload this form, fully completed, to the Journals Editorial Office. The Journal and/or Publisher will consider the information you have provided to decide whether to approve the proposed change in authorship. We may decide to contact your institution for more information or undertake a further investigation, if appropriate, before making a final decision.

SPRINGER NATURE

Change of authorship request form - Journals (pre-acceptance)

Section 1: Please provide the current title of manuscript

Manuscript ID no.: CDDISCOVERY-21-2112R

Title: RCN1 induces sorafenib resistance and malignancy in hepatocellular carcinoma by activating c-MYC signaling via the IRE1 α – XBP1s pathway

Section 2: Please provide the previous authorship, in the order shown on the manuscript before the changes were introduced. Please indicate the corresponding author by adding (CA) behind the name.

	First name(s)	Family name	ORCID or SCOPUS id, if available
1st author	Jia-Wei	Wang	
2nd author	Song	Wei	
3rd author	Xiao-Jun	Yang	
4th author	Shi-Pei	Qiu	
5th author	Xu	Lu	
6th author	Hao	Peng	
7th author	Lei	Li	
8th author	Bao-Lin	Wang(CA)	
9th author			
10th author			

Please use an additional sheet if there are more than 10 authors.

SPRINGER NATURE

Change of authorship request form - Journals (pre-acceptance)

Section 3: Please provide a justification for change. Please use this section to explain your reasons for changing the authorship of your manuscript, e.g. what necessitated the change in authorship? Please refer to the (journal) policy pages for more information about authorship. Please explain why omitted authors were not originally included and/or why authors were removed on the submitted manuscript.

In the revision, reviewers gave us a lot of comments to improve our manuscript. So we invited Ya-Qing Zhu, Li Ma and Yuan Liang help us revise the figures and make a careful language check again. For the principle of fairness, we need to change the current manuscript's author order according to their scientific contribution after the revision. In addition, We have acknowledged Lei Li for his great efforts in another manuscript and he had agreed with the rearrangement of the names.

Section 4: Proposed new authorship. Please provide your new authorship list in the order you would like it to appear on the manuscript. Please indicate the corresponding author by adding (CA) behind the name. <u>If the Corresponding Author has changed, please indicate the reason under section 3</u>.

	First name(s)	Family name (this name will appear in full on the final publication and will be searchable in various abstract and indexing databases)	Affiliated institute	E-mail address
1st author	Jia-Wei	Wang	The Second Affiliated Hospital of Nanjing Medical University	1307743510@qq.com
2nd author	Li	Ma	The Second Affiliated Hospital of Nanjing Medical University	ym741523@njmu.edu.cn
3rd author	Yuan	Liang	Nanjing Medical University	liangyuan@njmu.edu.cn
4th author	Xiao-Jun	Yang	The Second Affiliated Hospital of Nanjing Medical University	yangxiaojunvip@163.com
5th author	Song	Wei	The Second Affiliated Hospital of Nanjing Medical University	weisong5660@163.com
6th author	Hao	Peng	The Second Affiliated Hospital of Nanjing Medical University	Ljdzz1626@qq.com
7th author	Shi-Pei	Qiu	The Second Affiliated Hospital of Nanjing Medical University	qiushipei@njmu.edu.cn
8th author	Xu	Lu	The Second Affiliated Hospital of Nanjing Medical University	1179078611@qq.com
9th author	Ya-Qing	Zhu(CA)	the First Affiliated Hospital of Guangzhou University of Traditional Chinese Medicine	doctorzhuyq@163.com
10th author	Bao-Lin	Wang(CA)	The Second Affiliated Hospital of Nanjing Medical University	wangbl@njmu.edu.cn

Please use an additional sheet if there are more than 10 authors.

SPRINGER NATURE Springer Nature is one of the world's leading global research, educational and professional publishers, created in May 2015 through the combination of Nature Publishing Group, Palgrave Macmillan, Macmillan Education and Springer Science+Business Media.

SPRINGER NATURE

Change of authorship request form - Journals (pre-acceptance)

Section 5: Author contribution, Acknowledgement and Disclosures. Please use this section to provide a new disclosure statement and, if appropriate, acknowledge any contributors who have been removed as authors and ensure you state what contribution any new authors made (if applicable per the journal or book (series) policy). Please ensure these are updated in your manuscript - after approval of the change(s) - as our production department will not transfer the information in this form to your manuscript.

New acknowledgements:

None

New Disclosures (financial and non-financial interests, funding):

None

New Author Contributions statement (if applicable per the journal policy):

Ya-Qing Zhu critically reviewed and revised the final manuscript; Yuan Liang did the bioinformation analysis. Yuan Liang helped Jia-Wei Wang to draft the initial manuscript. Li Ma assisted with the experiments.

State 'Not applicable' if there are no new authors.

SPRINGER NATURE Springer Nature is one of the world's leading global research, educational and professional publishers, created in May 2015 through the combination of Nature Publishing Group, Palgrave Macmillan, Macmillan Education and Springer Science+Business Media.

SPRINGER NATURE

Change of authorship request form - Journals (pre-acceptance)

Section 6: Declaration of agreement. *All* authors, unchanged, new and removed *must* sign this declaration.
(NB: Please print the form, (docu)-sign and return/upload a scanned copy. Please note that signatures that have been inserted as an image file are acceptable as long as it is handwritten. Typed names in the signature box are unacceptable.) *** Please delete as appropriate. Delete all of the bold if you were on the original authorship list and are remaining as an author.**

	First name	Family name		Signature	Date
1st author	Jia-Wei	Wang	I agree to the proposed new authorship shown in section 4 ~~/and the addition/removal*of my name to the authorship list~~ /and the proposed change in corresponding author	Jiawei Wang	2021.10.05
2nd author	Li	Ma	I agree to the proposed new authorship shown in section 4 **/and the addition/~~removal*of my~~ ~~name to the~~ authorship list** /and the proposed change in corresponding author	Li Ma	2021.10.05
3rd author	Yuan	Liang	I agree to the proposed new authorship shown in section 4 **/and the addition/~~removal*~~of my name to the authorship list** /and the proposed change in corresponding author	Yuan Liang	2021.10.05
4th authors	Xiao-Jun	Yang	I agree to the proposed new authorship shown in section 4 ~~/and the addition/removal*of my name to the authorship list~~ /and the proposed change in corresponding author	Xiao-Jun Yang	2021.10.05
5th author	Song	Wei	I agree to the proposed new authorship shown in section 4 ~~/and the addition/removal*of my name to the authorship list~~ /and the proposed change in corresponding author	Song wei	2021.10.05
6th author	Hao	Peng	I agree to the proposed new authorship shown in section 4 ~~/and the addition/removal*of my name to the authorship list~~ /and the proposed change in corresponding author	Hao Peng	2021.10.05
7th author	Shi-Pei	Qiu	I agree to the proposed new authorship shown in section 4 ~~/and the addition/removal*of my name to the authorship list~~ /and the proposed change in corresponding author	Shi-Pei Qiu	2021.10.05

SPRINGER NATURE Springer Nature is one of the world's leading global research, educational and professional publishers, created in May 2015 through the combination of Nature Publishing Group, Palgrave Macmillan, Macmillan Education and Springer Science+Business Media.

SPRINGER NATURE

Change of authorship request form - Journals (pre-acceptance)

	First name	Family name		Signature	Date
8th author	Lei	Li	I agree to the proposed new authorship shown in section 4 /and ~~the addition~~/removal*of my name to the authorship list /and the proposed change in corresponding author	Lei Li	2021.10.05
9th author	Xu	Lu	I agree to the proposed new authorship shown in section 4 /~~and the addition/removal*of my name to the authorship list~~ /and the proposed change in corresponding author	Xu Lu	2021.10.05
10th author	Ya-Qing	Zhu	I agree to the proposed new authorship shown in section 4 /and the addition/~~removal*of~~ my name to the authorship list /and the proposed change in corresponding author	Yaqing Zhu	2021.10.05

Please use an additional sheet if there are more than 10 authors.

In case of author collaborations with formal agreement:

	Name of consortium/consortia	First name	Family name		Signature	Date
Representative/legal guarantor				I agree to the proposed new authorship shown in section 4 **/and the addition/removal*of my name to the authorship list** /and the proposed change in corresponding author		

Both added/removed authors should complete the information in the first table under Section 6.

---- End of form ----

SPRINGER NATURE Springer Nature is one of the world's leading global research, educational and professional publishers, created in May 2015 through the combination of Nature Publishing Group, Palgrave Macmillan, Macmillan Education and Springer Science+Business Media.

SPRINGER NATURE # Change of authorship request form - Journals (pre-acceptance)

	First name	Family name		Signature	Date
8th author	Bao-Lin	Wang	I agree to the proposed new authorship shown in section 4 ~~/and the addition/removal*of my name to the authorship list~~ /and the proposed change in corresponding author	Bao-Lin Wang	2021.10.05
9th author			I agree to the proposed new authorship shown in section 4 **/and the addition/removal*of my name to the authorship list** /and the proposed change in corresponding author		
10th author			I agree to the proposed new authorship shown in section 4 **/and the addition/removal*of my name to the authorship list** /and the proposed change in corresponding author		

Please use an additional sheet if there are more than 10 authors.

In case of author collaborations with formal agreement:

	Name of consortium/consortia	First name	Family name		Signature	Date
Representative/legal guarantor				I agree to the proposed new authorship shown in section 4 **/and the addition/removal*of my name to the authorship list** /and the proposed change in corresponding author		

Both added/removed authors should complete the information in the first table under Section 6.

---- End of form ----

SPRINGER NATURE Springer Nature is one of the world's leading global research, educational and professional publishers, created in May 2015 through the combination of Nature Publishing Group, Palgrave Macmillan, Macmillan Education and Springer Science+Business Media.

Re: Changes to authorship

"吕凌课题组学生王家伟" <1307743510@qq.com>

To: "汪宝林" <wangbl@njmu.edu.cn>

Date: 2021-10-5 13:40:28

Att:

Confirmed

发自我的iPhone

------------------ Original ------------------

From: 汪宝林 <wangbl@njmu.edu.cn>
Date: Tue,Oct 5,2021 1:27 PM
To: Song Wei <weisong5660@163.com>, Jia-Wei Wang <1307743510@qq.com>, Ya-Qing Zhu <doctorzhuyq@163.com>, Lei Li <2941921584@qq.com>, Xu Lu <1179078611@qq.com>, Shi-Pei Qiu <qiushipei@njmu.edu.cn>, Hao Peng <Ljdzz1626@qq.com>, Xiao-Jun Yang <yangxiaojunvip@163.com>, Yuan Liang <liangyuan@njmu.edu.cn>, Li Ma <ym741523@njmu.edu.cn>
Subject: Re: Changes to authorship

Manuscript Number: CDDISCOVERY-21-2112R

Title: RCN1 induces sorafenib resistance and malignancy in hepatocellular carcinoma by activating c-MYC signaling via the IRE1α–XBP1s pathway.

Authors: Jia–Wei Wang · Li Ma · Yuan Liang · Xiao–Jun Yang · Song Wei · Hao Peng ·Shi–Pei Qiu · Xu Lu · Ya–Qing Zhu & Bao–Lin Wang

Corresponding authors:Ya–Qing Zhu or Bao–Lin Wang

Please reply as soon as possible.

Re: Changes to authorship

2941921584 <2941921584@qq.com>
To: "汪宝林" <wangbl@njmu.edu.cn>
Date: 2021-10-5 13:34:33
Att:

Confirmed

发自我的iPhone

------------------ Original ------------------

From: 汪宝林 <wangbl@njmu.edu.cn>
Date: Tue,Oct 5,2021 1:27 PM
To: Song Wei <weisong5660@163.com>, Jia-Wei Wang <1307743510@qq.com>, Ya-Qing Zhu <doctorzhuyq@163.com>, Lei Li <2941921584@qq.com>, Xu Lu <1179078611@qq.com>, Shi-Pei Qiu <qiushipei@njmu.edu.cn>, Hao Peng <Ljdzz1626@qq.com>, Xiao-Jun Yang <yangxiaojunvip@163.com>, Yuan Liang <liangyuan@njmu.edu.cn>, Li Ma <ym741523@njmu.edu.cn>
Subject: Re: Changes to authorship

Manuscript Number: CDDISCOVERY-21-2112R

Title: RCN1 induces sorafenib resistance and malignancy in hepatocellular carcinoma by activating c-MYC signaling via the IRE1α–XBP1s pathway.

Authors: Jia–Wei Wang · Li Ma · Yuan Liang · Xiao–Jun Yang · Song Wei · Hao Peng ·Shi–Pei Qiu · Xu Lu · Ya–Qing Zhu & Bao–Lin Wang

Corresponding authors:Ya–Qing Zhu or Bao–Lin Wang

Please reply as soon as possible.

Re: Changes to authorship

1179078611 <1179078611@qq.com>

To: "汪宝林" <wangbl@njmu.edu.cn>

Date: 2021-10-5 13:48:33

Att:

Confirmed

---Original---

From: "汪宝林"<wangbl@njmu.edu.cn>
Date: Tue, Oct 5, 2021 13:27 PM
To: "Song Wei"<weisong5660@163.com>;"Jia-Wei Wang"<1307743510@qq.com>;"Ya-Qing Zhu" <doctorzhuyq@163.com>;"Lei Li"<2941921584@qq.com>;"Xu Lu"<1179078611@qq.com>;"Shi-Pei Qiu" <qiushipei@njmu.edu.cn>;"Hao Peng"<Ljdzz1626@qq.com>;"Xiao-Jun Yang" <yangxiaojunvip@163.com>;"Yuan Liang"<liangyuan@njmu.edu.cn>;"Li Ma"<ym741523@njmu.edu.cn>;
Subject: Changes to authorship

Manuscript Number: CDDISCOVERY-21-2112R

Title: RCN1 induces sorafenib resistance and malignancy in hepatocellular carcinoma by activating c-MYC signaling via the IRE1α–XBP1s pathway.

Authors: Jia–Wei Wang・Li Ma・Yuan Liang・Xiao–Jun Yang・Song Wei・Hao Peng・Shi–Pei Qiu・Xu Lu・Ya–Qing Zhu & Bao–Lin Wang

Corresponding authors:Ya–Qing Zhu or Bao–Lin Wang

Please reply as soon as possible.

Re: Changes to authorship

"彭颢" <ljdzz1626@qq.com>

收件人："汪宝林" <wangbl@njmu.edu.cn>

时　间：2021-10-5 13:33:49

附　件：

Agree to these changs

发自我的iPhone

------------------ Original ------------------

From: 汪宝林 <wangbl@njmu.edu.cn>
Date: Tue,Oct 5,2021 1:27 PM
To: Song Wei <weisong5660@163.com>, Jia-Wei Wang <1307743510@qq.com>, Ya-Qing Zhu <doctorzhuyq@163.com>, Lei Li <2941921584@qq.com>, Xu Lu <1179078611@qq.com>, Shi-Pei Qiu <qiushipei@njmu.edu.cn>, Hao Peng <Ljdzz1626@qq.com>, Xiao-Jun Yang <yangxiaojunvip@163.com>, Yuan Liang <liangyuan@njmu.edu.cn>, Li Ma <ym741523@njmu.edu.cn>
Subject: Re: Changes to authorship

Manuscript Number: CDDISCOVERY-21-2112R

Title: RCN1 induces sorafenib resistance and malignancy in hepatocellular carcinoma by activating c-MYC signaling via the IRE1α–XBP1s pathway.

Authors: Jia–Wei Wang・Li Ma・Yuan Liang・Xiao–Jun Yang・Song Wei・Hao Peng・Shi–Pei Qiu・Xu Lu・Ya–Qing Zhu & Bao–Lin Wang

Corresponding authors:Ya–Qing Zhu or Bao–Lin Wang

Please reply as soon as possible.

Re:Changes to authorship

"梁渊" <liangyuan@njmu.edu.cn>

To: "汪宝林" <wangbl@njmu.edu.cn>

Cc: Song Wei <weisong5660@163.com>, Jia-Wei Wang <1307743510@qq.com>, Ya-Qing Zhu <doctorzhuyq@163.com>, Lei Li <2941921584@qq.com>, Xu Lu <1179078611@qq.com>, Shi-Pei Qiu <qiushipei@njmu.edu.cn>, Hao Peng <Ljdzz1626@qq.com>, Xiao-Jun Yang <yangxiaojunvip@163.com>, Li Ma <ym741523@njmu.edu.cn>

Date: 2021-10-5 13:31:24

Att:

---

confirmed

发件人："汪宝林" <wangbl@njmu.edu.cn>
发送日期：2021-10-05 13:27:53
收件人：Song Wei <weisong5660@163.com>,Jia-Wei Wang <1307743510@qq.com>,Ya-Qing Zhu <doctorzhuyq@163.com>,Lei Li <2941921584@qq.com>,Xu Lu <1179078611@qq.com>,Shi-Pei Qiu <qiushipei@njmu.edu.cn>,Hao Peng <Ljdzz1626@qq.com>,Xiao-Jun Yang <yangxiaojunvip@163.com>,Yuan Liang <liangyuan@njmu.edu.cn>,Li Ma <ym741523@njmu.edu.cn>
主题：Changes to authorship

> Authors: Jia-Wei Wang · Li Ma · Yuan Liang · Xiao-Jun Yang · Song Wei · Hao Peng ·Shi-Pei Qiu · Xu Lu · Ya-Qing Zhu & Bao-Lin Wang
>
> Corresponding authors:Ya-Qing Zhu or Bao-Lin Wang
>
> Please reply as soon as possible.

Re:Changes to authorship

ym741523@njmu.edu.cn

To: "汪宝林" <wangbl@njmu.edu.cn>

Cc: Song Wei <weisong5660@163.com>, Jia-Wei Wang <1307743510@qq.com>, Ya-Qing Zhu <doctorzhuyq@163.com>, Lei Li <2941921584@qq.com>, Xu Lu <1179078611@qq.com>, Shi-Pei Qiu <qiushipei@njmu.edu.cn>, Hao Peng <Ljdzz1626@qq.com>, Xiao-Jun Yang <yangxiaojunvip@163.com>, Yuan Liang <liangyuan@njmu.edu.cn>

Date: 2021-10-5 13:33:26

Att:

---

confirmed

在 2021-10-05 13:27:53，"汪宝林" <wangbl@njmu.edu.cn> 写道：

Re: Changes to authorship

"魏菘" <weisong5660@163.com>

To: "汪宝林" <wangbl@njmu.edu.cn>

Date: 2021-10-5 13:34:59

Att:

confirmed

---- Replied Message ----

From	汪宝林<wangbl@njmu.edu.cn>
Date	10/05/2021 13:27
To	Song Wei<weisong5660@163.com>,<br>Jia-Wei Wang<1307743510@qq.com>,<br>Ya-Qing Zhu<doctorzhuyq@163.com>,<br>Lei Li<2941921584@qq.com>,<br>Xu Lu<1179078611@qq.com>,<br>Shi-Pei Qiu<qiushipei@njmu.edu.cn>,<br>Hao Peng<ljdzz1626@qq.com>,<br>Xiao-Jun Yang<yangxiaojunvip@163.com>,<br>Yuan Liang<liangyuan@njmu.edu.cn>,<br>Li Ma<ym741523@njmu.edu.cn>
Subject	Changes to authorship

Authors: Jia-Wei Wang · Li Ma · Yuan Liang · Xiao-Jun Yang · Song Wei · Hao Peng ·Shi-Pei Qiu · Xu Lu · Ya-Qing Zhu & Bao-Lin Wang

Corresponding authors:Ya-Qing Zhu or Bao-Lin Wang

Please reply as soon as possible.

Re: Changes to authorship

yangxiaojun <yangxiaojunvip@163.com>

To: "汪宝林" <wangbl@njmu.edu.cn>

Date: 2021-10-5 13:45:51

Att:

Confirmed

---- Replied Message ----

From	汪宝林<wangbl@njmu.edu.cn>
Date	10/05/2021 13:27
To	Song Wei<weisong5660@163.com>,<br>Jia-Wei Wang<1307743510@qq.com>,<br>Ya-Qing Zhu<doctorzhuyq@163.com>,<br>Lei Li<2941921584@qq.com>,<br>Xu Lu<1179078611@qq.com>,<br>Shi-Pei Qiu<qiushipei@njmu.edu.cn>,<br>Hao Peng<ljdzz1626@qq.com>,<br>Xiao-Jun Yang<yangxiaojunvip@163.com>,<br>Yuan Liang<liangyuan@njmu.edu.cn>,<br>Li Ma<ym741523@njmu.edu.cn>
Subject	Changes to authorship

Authors: Jia-Wei Wang · Li Ma · Yuan Liang · Xiao-Jun Yang · Song Wei · Hao Peng ·Shi-Pei Qiu · Xu Lu · Ya-Qing Zhu & Bao-Lin Wang

Corresponding authors:Ya-Qing Zhu or Bao-Lin Wang

Please reply as soon as possible.

Re: Changes to authorship

"朱亚青" <doctorzhuyq@163.com>

To: "汪宝林" <wangbl@njmu.edu.cn>

Date: 2021-10-5 13:39:22

Att:

Confirmed

---- Replied Message ----

From	汪宝林<wangbl@njmu.edu.cn>
Date	10/05/2021 13:27
To	Song Wei<weisong5660@163.com>,<br>Jia-Wei Wang<1307743510@qq.com>,<br>Ya-Qing Zhu<doctorzhuyq@163.com>,<br>Lei Li<2941921584@qq.com>,<br>Xu Lu<1179078611@qq.com>,<br>Shi-Pei Qiu<qiushipei@njmu.edu.cn>,<br>Hao Peng<ljdzz1626@qq.com>,<br>Xiao-Jun Yang<yangxiaojunvip@163.com>,<br>Yuan Liang<liangyuan@njmu.edu.cn>,<br>Li Ma<ym741523@njmu.edu.cn>
Subject	Changes to authorship

Authors: Jia-Wei Wang · Li Ma · Yuan Liang · Xiao-Jun Yang · Song Wei · Hao Peng ·Shi-Pei Qiu · Xu Lu · Ya-Qing Zhu & Bao-Lin Wang

Corresponding authors:Ya-Qing Zhu or Bao-Lin Wang

Please reply as soon as possible.

Re:Changes to authorship

"邱诗沛" <qiushipei@njmu.edu.cn>

收件人："汪宝林" <wangbl@njmu.edu.cn>

时　间：2021-10-5 16:12:59

附　件：

confirmed

发件人："汪宝林" <wangbl@njmu.edu.cn>
发送日期：2021-10-05 13:27:53
收件人：Song Wei <weisong5660@163.com>,Jia-Wei Wang <1307743510@qq.com>,Ya-Qing Zhu <doctorzhuyq@163.com>,Lei Li <2941921584@qq.com>,Xu Lu <1179078611@qq.com>,Shi-Pei Qiu <qiushipei@njmu.edu.cn>,Hao Peng <Ljdzz1626@qq.com>,Xiao-Jun Yang <yangxiaojunvip@163.com>,Yuan Liang <liangyuan@njmu.edu.cn>,Li Ma <ym741523@njmu.edu.cn>
主题：Changes to authorship

> Authors: Jia-Wei Wang · Li Ma · Yuan Liang · Xiao-Jun Yang · Song Wei · Hao Peng ·Shi-Pei Qiu · Xu Lu · Ya-Qing Zhu & Bao-Lin Wang
>
> Corresponding authors:Ya-Qing Zhu or Bao-Lin Wang
>
> Please reply as soon as possible.
