# Supplementary material for: RCN1 induces sorafenib resistance and malignancy in hepatocellular carcinoma by activating c-MYC signaling via the IRE1α–XBP1s pathway
Source: Cell Death Discov. 2021 Oct 18;7:298. doi: 10.1038/s41420-021-00696-6 (PMC8523720; doi:10.1038/s41420-021-00696-6)
Supplement: Supplementary file 3 — Attribution of Authorship [file 41420_2021_696_MOESM3_ESM.pdf]

DECLARATION OF CONTRIBUTIONS TO ARTICLE

ADMC

Manuscript Number:	Journal Name:	
CDDISCOVERY-21-2112R	*Cell Death Discovery*	(the 'Journal')

Proposed Title of the Contribution:

RCN1 induces sorafenib resistance and malignancy in hepatocellular carcinoma by activating c-MYC signaling via the IRE1α–XBP1s pathway (the 'Contribution)

Author(s):

Jia-Wei Wang · Li Ma · Yuan Liang · Xiao-Jun Yang · Song Wei · Hao Peng ·Shi-Pei Qiu · Xu Lu · Ya-Qing Zhu & Bao-Lin Wang (the 'Authors')

For all *CDDis*covery articles, each person named as an author in the published version must be able to show he or she has contributed substantially to the article.

Authorship credit should be based on 1) substantial contributions to conception and design, acquisition of data, or analysis and interpretation of data; 2) drafting the article or revising it critically for important intellectual content; and 3) final approval of the version to be published. Authors should meet conditions 1, 2 and 3.

Any person who cannot be shown to have made a substantial contribution to the article cannot be listed as an author in the final version. The name of any person who is deemed to have made a minor contribution can, however, appear in the Acknowledgments section of the article.

Please complete the table below to indicate the contributions of all named authors to the manuscript.

Author Full Name:	Specification of Contribution to the Manuscript:
Jia-Wei Wang	performed the experiments, analyzed the data and wrote the first draft of manuscript
Li Ma	performed the experiments, analyzed the data and participated critical discussion
Yuan Liang	performed the experiments, analyzed the data and participated critical discussion
Hao Peng	performed the experiments, analyzed the data and participated critical discussion
Xiao-Jun Yang	performed the experiments, analyzed the data and participated critical discussion
Shi-Pei Qiu	performed the experiments, analyzed the data and participated critical discussion
Xu Lu	performed the experiments, analyzed the data and participated critical discussion
Song Wei	assisted with the experiment and provided technical help
Ya-Qing Zhu	reviewed and revised the final manuscript
Bao-Lin Wang	designed and supervised the research

Please complete the table below to indicate the contributions of all named authors to the figures.

Figure 1:

Jia-Wei Wang and Yuan Liang generated the data and prepared panels A-K.Yuan Liang did the bioinformation analysis. Xu Lu assisted with the experiments.

Figure 2:

Jia-Wei Wang ,Song Wei and Li Ma generated the data and prepared panels A-I.Xu Lu and Xiao-JunYang assisted with the experiments.

Figure 3:

Jia-Wei Wang generated the data and prepared panels A-E. Shi-Pei Qiu and Hao Peng assisted with the experiments.

Figure 4:

Jia-Wei Wang and Hao Peng generated the data and assembled the panels A-F.

Figure 5:

Jia-Wei Wang and Xu Lu generated the data and prepared panels A-E. Hao Peng assisted with the experiments.

Figure 6:

Jia-Wei Wang and Shi-Pei Qiu generated the data and assemled the panels A-H.Yuan Liang did the bioinformation analysis.

Signed for and on behalf of the Author(s):	Print Name:	Date:
Baolin Wang	Bao-Lin Wang	2021-10-01
